# Factors Associated With Burnout and Stress in Trainee Physicians: A Systematic Review and Meta-analysis

**DOI:** 10.1001/jamanetworkopen.2020.13761

**Published:** 2020-08-18

**Authors:** Anli Yue Zhou, Maria Panagioti, Aneez Esmail, Raymond Agius, Martie Van Tongeren, Peter Bower

**Affiliations:** 1 Division of Population Health, Health Services Research & Primary Care, University of Manchester, Manchester, United Kingdom; 2 National Institute for Health Research School for Primary Care Research, Division of Population Health, Health Services Research & Primary Care, University of Manchester, Manchester, United Kingdom

## Abstract

**Importance:**

Evidence suggests that physicians experience high levels of burnout and stress and that trainee physicians are a particularly high-risk group. Multiple workplace- and non–workplace-related factors have been identified in trainee physicians, but it is unclear which factors are most important in association with burnout and stress. Better understanding of the most critical factors could help inform the development of targeted interventions to reduce burnout and stress.

**Objective:**

To estimate the association between different stressors and burnout/stress among physicians engaged in standard postgraduate training (ie, trainee physicians).

**Data Sources:**

Medline, Embase, PsycINFO, and Cochrane Database of Systematic reviews from inception until April 30, 2019. Search terms included *trainee*, *foundation year*, *registrar*, *resident*, and *intern*.

**Study Selection:**

Studies that reported associations between stressors and burnout/stress in trainee physicians.

**Data Extraction and Synthesis:**

Two independent reviewers extracted the data and assessed the quality of the evidence. The main meta-analysis was followed by sensitivity analyses. All analyses were performed using random-effects models, and heterogeneity was quantified using the *I*^2^ statistic.

**Main Outcome and Measures:**

The main outcome was the association between burnout/stress and workplace- or non–workplace-related factors reported as odds ratios (ORs) and their 95% CIs.

**Results:**

Forty-eight studies were included in the meta-analysis (n = 36 266, median age, 29 years [range, 24.6-35.7 years]). One study did not specify participants’ sex; of the total population, 18 781 participants (52%) were men. In particular, work demands of a trainee physician were associated with a nearly 3-fold increased odds for burnout/stress (OR, 2.84; 95% CI, 2.26-3.59), followed by concerns about patient care (OR, 2.35; 95% CI, 1.58-3.50), poor work environment (OR, 2.06; 95% CI, 1.57-2.70), and poor work-life balance (OR, 1.93; 95% CI, 1.53-2.44). Perceived/reported poor mental or physical health (OR, 2.41; 95% CI, 1.76-3.31), female sex (OR, 1.34; 95% CI, 1.20-1.50), financial worries (OR, 1.35; 95% CI, 1.07-1.72), and low self-efficacy (OR, 2.13; 95% CI, 1.31-3.46) were associated with increased odds for burnout/stress, whereas younger age and a more junior grade were not significantly associated.

**Conclusions and Relevance:**

The findings of this study suggest that the odds ratios for burnout and stress in trainee physicians are higher than those for work-related factors compared with nonmodifiable and non–work-related factors, such as age and grade. These findings support the need for organizational interventions to mitigate burnout in trainee physicians.

## Key Points

**Question:**

What factors are associated with burnout/stress in trainee physicians?

**Findings:**

This systematic review and meta-analysis of 48 studies included 36 266 trainee physicians. The odds ratios for the associations between workplace factors and burnout/stress were found to be higher compared with nonmodifiable and non–work-related factors such as age and grade.

**Meaning:**

The findings of this study highlight the importance of improving training and work environments to possibly prevent burnout among trainee physicians and suggest that implementing multicomponent interventions to target major stressors uncovered in this study could be promising.

## Teaser

This systematic review and meta-analysis examines the factors associated with burnout and stress among physicians receiving standard postgraduate training.

## Introduction

Trainee physicians are qualified physicians engaged in postgraduate training.^1^ There is evidence suggesting that physicians experience high levels of burnout and stress, and trainee physicians are a particularly high-risk group.^2,3,4,5^ Stress is a state of mental strain resulting from demanding circumstances.^6^ Burnout consists of 3 components: emotional exhaustion, reduced sense of personal accomplishment, and depersonalization.^7,8^ High burnout and stress levels have been found in trainee physicians working in the US, Australia, and Canada.^9,10,11,12,13^ Surveys on trainee physicians suggest that 50% were experiencing burnout symptoms and 80% were experiencing high stress.^14^ Burnout in trainee physicians can have profound effects on personal well-being, career prospects, and relationships and may jeopardize patient care.^14^ The well-being of trainee physicians is a benchmark for the sustainability of health care systems.^15,16^ Better understanding of factors that underpin feelings of stress and burnout in trainee physicians has important implications.

Workplace-related factors, such as workload and work-life conflict, and non–work-related factors have been found to be associated with burnout.^13,16,17^ However, owing to variations in methods and presentation of results, it is difficult to compare the findings between published studies and explore reasons for inconsistent results. Thus, we have conducted the first systematic review and meta-analysis to identify workplace- and non–workplace-related factors that are associated with burnout/stress in trainee physicians and the relative importance of these factors.

## Methods

This review is reported in line with the Preferred Reporting Items for Systematic Reviews and Meta-Analyses (PRISMA) guidelines and Meta-analyses of Observational Studies in Epidemiology (MOOSE) guidelines. MOOSE guidelines were also adopted because PRISMA mainly focuses on intervention studies whereas MOOSE guidelines focus on observational studies.^18,19^

Medline, Embase, PsycINFO, and the Cochrane Database of Systematic reviews were searched from inception until April 30, 2019. The search strategy included combinations of 3 key blocks of terms (*stress*, *trainee physicians*, and *determinants of stress*) using a combination of Medical Subject Headings and text words (eTable 1 in the Supplement). We used a wide range of terms for trainee physicians in our search, including *trainee*, *foundation year*, *registrar*, *resident*, and *intern*.

Database searches were supplemented by manual searches of reference lists of included articles. No previous systematic reviews were identified in the literature or within PROSPERO. eMethods in the Supplement provides the systematic review protocol.

### Eligibility Criteria

Studies were eligible for inclusion if they met the following criteria. Regarding population, qualified physicians who were engaged in standard postgraduate training (ie, trainee physicians) were included. Studies that were based on a mix of trainee physicians and other physicians or health professionals were included if trainee physicians composed at least 70% of the sample.

Workplace-related factors (eg, work demands), non–workplace-related factors (eg, poor health), and demographic characteristics that may be associated with burnout and stress (eg, sex) were analyzed. In particular, studies had to explicitly state that they examined factors associated with burnout/stress (a wide range of terms was used, including *determinants*, *drivers*, *contributors*, *drivers*, *causes*, *predictor*, *risk*, or *associate*) in titles, abstracts, or key words (eMethods in the Supplement), and the main outcomes of the study were required to be burnout and stress. Studies using quantitative research designs, such as observational (eg, cohort, cross-sectional, and case-control), were included in the meta-analysis. Studies that took place in any health care setting, including primary and hospital care, were considered eligible.

Studies were excluded if they had not explicitly focused on burnout or stress, such as those exploring the determinants of specific psychiatric condition criteria (eg, depression and generalized anxiety disorder) or did not report investigation of factors associated with burnout or stress. Other exclusion criteria were studies reported as gray literature (research published outside the traditional academic literature), conference abstracts, letters to the editor, and non–peer-reviewed investigations, as well as those not published in the English language. In addition, studies that did not provide data amenable to meta-analysis were excluded.

### Data Selection

Searches were exported into EndNote (Clarivate Analytics), and duplicate studies were removed. Study selection involved 2 stages. First, titles and abstracts of the identified studies were screened; subsequently, the full texts of relevant studies were accessed and further screened against the eligibility criteria. The title and abstract screening was undertaken by 1 of us (A.Y.Z.), and full text screening was performed by 2 of us (A.Y.Z. and M.P.). Interrater reliability was high (κ = 0.84). Disagreements were resolved through discussions.

If necessary, we contacted authors of relevant articles to request full texts or additional data. An Excel-based extraction form was piloted on 5 randomly selected studies. Data on the following factors were extracted: (1) country, method of recruitment, health care setting, research design, control, and location of the study; (2) sample size, age, sex, specialty, and trainee physician grade of the population; and (3) factors associated with burnout/stress (burnout, stress, and other), types of analysis used, and type of factors identified as outcomes.

We used the Newcastle-Ottawa Scale (NOS) to critically appraise the quality of the studies. This scale was designed to assess the quality of nonrandomized studies (eg, case-control),^20^ but has been adapted for undertaking critical appraisals of cross-sectional studies.^21^ This modified NOS instrument provides scores from 0 to 10, with studies scoring greater than or equal to 6 classified as high quality. Two of us (A.Y.Z. and M.P.) assessed 20% of the studies, and interrater reliability was high (κ = 0.93). Subsequent articles were assessed by 1 of us (A.Y.Z.).

### Statistical Analysis

The primary outcome of this review was the association of identified factors with burnout/stress in trainee physicians. We calculated odds ratios (ORs) together with 95% CIs using Comprehensive Meta-analysis software (Biostat).^22^ Pooled ORs and forest plots were computed using the metaan command in Stata, version 14 (StataCorp).^23^ We chose to use ORs to pool the results because this measure was most commonly applied in individual studies and because ORs are considered more appropriate for cross-sectional studies.^24^ In accordance with recommendations,^22^ across studies reporting multiple measures of the same stressor category (eg, different measures of job demands, such as time on call or long working hours), the median ORs were computed to ensure that each study contributed only 1 estimate to each analysis. The *I*^2^ statistic was used to assess heterogeneity between studies. An *I*^2^ value of 0% to 49% indicated low heterogeneity; 50% to 74%, moderate; and 75% to 100%, high.^25^

Three sensitivity analyses were performed to examine whether the results were robust by (1) only including highly rated methodologic studies in the analyses (NOS score ≥6), (2) only including studies using measures of burnout, and (3) only including studies using the Maslach Burnout Inventory,^7^ which is typically viewed as a measure of prolonged stress.

Potential for publication bias was assessed on all pooled outcomes that included 9 or more studies^26^ by inspecting the symmetry of funnel plots and using the Egger test.^27^ Funnel plots were constructed using the metafunnel command and the Egger test was computed using the metabias command.^28,29^ All analyses were performed in Stata, version 14. A 2-tailed *P* value <.05 was the level of significance.

## Results

**Figure 1.**
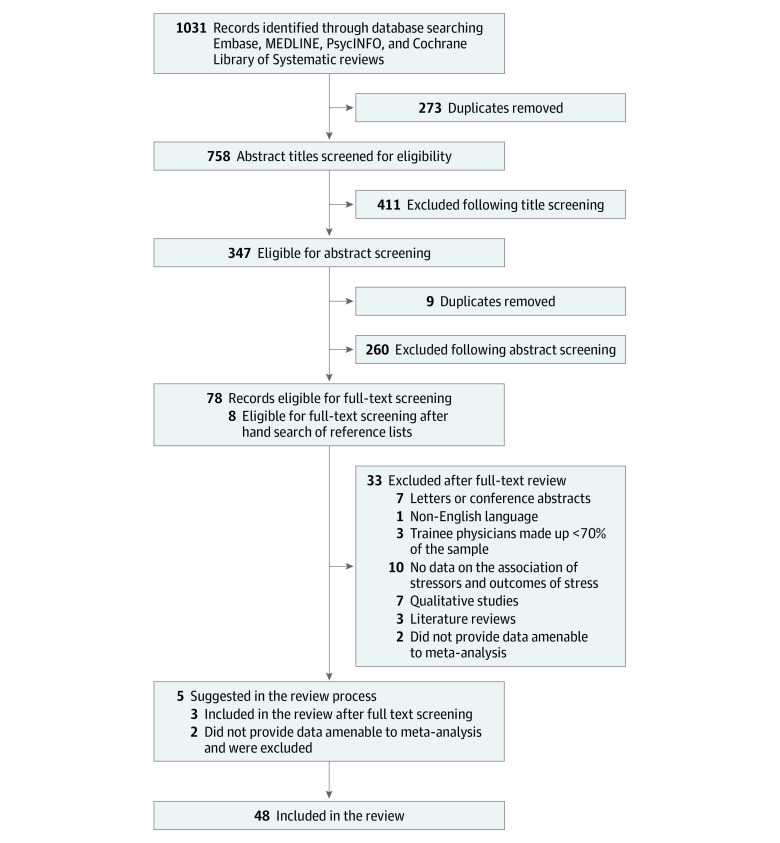
Flowchart of Studies Included in the Review

Overall, 1036 records were screened for eligibility. Following full-text screening, 48 studies met the eligibility criteria (Figure 1). Table 1 reports the included studies regarding population size, trainee grade, median age, study setting, location, types of measures used, response rates, and adapted NOS score. Across the 48 studies, a pooled cohort of 36 266 participants was formed.^4,10,11,12,13,17,30,31,32,33,34,35,36,37,38,39,40,41,42,43,44,45,46,47,48,49,50,51,52,53,54,55,56,57,58,59,60,61,62,63,64,65,66,67,68,69,70,71^ The median number of recruited trainee physicians was 203 (range, 58-16 394). One study^43^ did not specify participants’ sex; of the total cohort, 18 781 participants (52%) were men and 17 315 participants (48%) were women; median age was 29 years (range, 24.6-35.7). Thirty-seven studies^11,13,30,31,32,33,35,36,37,38,40,41,42,43,44,45,46,47,48,49,50,51,53,54,57,58,59,61,62,63,64,65,67,68,69,70,71^ (77%) used validated measures of burnout/stress. The Maslach Burnout Inventory was the most common measure of burnout (42%). The median response rate for cross-sectional studies was 61% (range, 15%-90%). Twenty-four studies^31,33,34,35,37,41,42,43,46,47,48,49,52,53,57,58,60,61,64,65,66,67,68,70^ (50%) had an adapted NOS score greater than or equal to 6 (range, 2-8). Eleven factors were identified in this review (Table 2).

**Table 1.** Characteristics of Studies, Populations, and Outcomes Included in the Review

Study	Country	Health care setting	Research design	Sample size	Men, %	Mean age, y	Specialties	Working experience	Measure of wellness^a^	Categories of stressors identified	Newcastle-Ottawa Scale Score
Abdulghani et al,^11^ 2015	Saudi Arabia	Hospital and primary care	Cross-sectional	318	60	27.9	Multiple	Residency year 1-4	2, Kessler-10 psychological distress instrument	Specialty grade, demographics, poor work-life balance, work demands	4
Abdulghani et al,^30^ 2014	Saudi Arabia	Hospital	Cross-sectional	404	63	Not stated	Multiple	Internship	2, Kessler-10 psychological distress instrument	Demographics, specialty	4
Afzal et al,^31^ 2010	US	Hospital and primary care	Cross-sectional	134	58	Not stated	Multiple	Residency	1, Maslach Burnout Inventory	Specialty, demographics, poor work-life balance	7
Al-Ma'mari et al,^32^ 2016	Canada	Hospital	Cross-sectional	47	13	Not stated	Obstetrics and gynecology	Residents	1, Maslach Burnout Inventory	Poor work environment	5
Antoniou et al,^33^ 2003	Greece	Public hospital, clinics	Cross-sectional	355	54	Age range, 25-42	Not specified	All training grades	2, Occupational Stress Index	Poor career development, poor work-life balance, poor work environment, personal and self-efficacy, concerns about patient care, work demands, financial worries	6
Baer et al,^34^ 2017	US	Public hospital	Cross-sectional	258	21	29	Pediatrics	Residents	1, 2-item burnout measure validated again Maslach Burnout Inventory	Demographics, perceived/reported poor mental or physical health poor work-life balance, work demands, seniority and grade, poor work environment	7
Baldwin et al,^35^ 1997	UK	Hospital	Prospective cohort	142	55	25	Not specified	Senior house officers	2, General Health Questionnaire	Work demands, concerns about patient care	6
Bellolio et al,^36^ 2014	US	Hospital	Cross-sectional	191	53	Not stated	Multiple	Residents	1, 2, Professional Quality of Life Scale which includes measures on burnout	Demographics, specialty, personal and self-efficacy, work demands	2
Blanchard et al,^37^ 2010	France	Hospital	Cross-sectional	204	60	Median, 28	Oncology and hematology	Residents	1, Maslach Burnout Inventory	Work demands, poor career development, concerns about patient care	8
Byrne et al,^38^ 2016	Ireland	Hospital	Cross-sectional	270	39	Not stated	Multiple	Internship	3, General Health Questionnaire	Poor career development, perceived/reported poor mental or physical health, poor work-life balance	5
Cohen and Patten,^10^ 2005	Canada	Hospital	Cross-sectional	415	47	29	Multiple	Residents	2, Sources and amount of perceived stress	Poor work-life balance, financial worries, work demands, poor work environment, perceived/reported poor mental or physical health	4
Cooke et al,^39^ 2013	Australia	Primary care	Cross-sectional	128	33	>20	General practice	Registrar level	1,3, Single-item scale for burnout validated against Maslach Burnout Inventory, professional quality-of-life scale	Concerns about patient care, seniority and grade, poor work environment, demographics, work demands, personal and self-efficacy, poor work-life balance, perceived/reported poor mental or physical health	5
Creed et al,^40^ 2014	Australia	Hospital and primary care	Cross-sectional	355	32	28	Multiple	<4 y of graduation	1,2, Copenhagen Burnout Inventory, 4-item academic stress scale	Work demands, financial worries, poor career development	5
Dyrbye et al,^41^ 2018	US	Hospital and primary care	Cross-sectional	3588	49	Not stated	Multiple	Residents	1, Maslach Burnout Inventory	Specialty, demographics, poor work-life balance, financial worries	8
Esan et al,^42^ 2014	Nigeria	Hospital	Cross-sectional	128	73	Not stated	Multiple	Residents	3, General Health Questionnaire	Demographics, financial worries, poor work environment, poor career development, work demands	6
Firth-Cozens,^43^ 1992	UK	Hospital	Cross-sectional	170	Not stated	Not stated	Multiple	Postgraduate year 1	3, General Health Questionnaire	Personal and self-efficacy, demographics, poor work environment, perceived/reported poor mental or physical health	6
Firth-Cozens,^44^ 1990	UK	Hospital	Cross-sectional	70	0	Not stated	Multiple	Postgraduate year 1	3, General Health Questionnaire	Poor work environment, concerns about patient care, poor work-life balance, work demands, poor career development, financial worries	4
Firth Cozens and Morrison,^45^ 1989	UK	Hospital	Cross-sectional	173	57	24.6	Multiple	Postgraduate year 1	3, General Health Questionnaire	Concerns about patient care, poor work environment, work demands	4
Galam et al,^46^ 2013	France	Primary care	Cross-sectional	169	53	25.4	General practice	General practice trainees	1, Maslach Burnout Inventory	Demographics, work demands, poor work environment, poor work-life balance, poor career development	6
Galam et al,^47^ 2017	France	Primary care	Longitudinal	173	31.3	26.4	General practice	General practice trainees	1, Maslach Burnout Inventory	Personal and self-efficacy	6
Gouveia et al,^48^ 2017	Brazil	Hospital	Cross-sectional	129	48	Not stated	Multiple	Residents	1, Maslach Burnout Inventory	Concerns about patient care, specialty	6
Guenette and Smith,^49^ 2017	US	Hospital	Cross-sectional	94	63	Not stated	Radiology	Residents	1, Maslach Burnout Inventory	Poor work-life balance, seniority, demographics	7
Hameed et al,^50^ 2018	Saudi Arabia	Hospital	Cross-sectional	181	41	27.6	Multiple	Residents	1, Maslach Burnout Inventory	Seniority, demographics, specialty	5
Hannan et al,^51^ 2018	Ireland	Hospital	Cross-sectional	101	44	28	Multiple	Interns	1,3, Maslach Burnout inventory and General Health Questionnaire	Poor work environment, poor career development, concerns about patient care, financial worries, perceived/reported poor mental or physical health, work demands	4
Haoka et al,^13^ 2010	Japan	Hospital	Cross-sectional	348	67	Men, 26.2; women, 25.6	Multiple	Residents	3, General Health Questionnaire	Perceived/reported poor mental or physical health, work demands, poor work-life balance, personal and self-efficacy	5
Jex et al,^52^ 1991	US	Hospital	Cross-sectional	1785	70	30	Multiple	Residents	2, General and work-related psychological strain	Work demands, concerns about patient care, perceived/reported poor mental or physical health	7
Kassam et al,^53^ 2015	Canada	University of Calgary trainees	Cross-sectional	317	39	30.9	Multiple	Residents	1,3, Copenhagen Burnout Inventory and work satisfaction	Work demands, personal and self-efficacy, perceived/reported poor mental or physical health	7
Kimo Takayesu et al,^54^2014	US	Residency program	Cross-sectional	218	59	Not stated	Emergency medicine	Residents	1, Maslach Burnout Inventory	Poor work environment, personal and self-efficacy	5
Kshatri et al,^55^ 2017	India	3 Hospitals	Cross-sectional	250	68	29	Multiple	Residents	2, Workplace Stress Scale	Seniority, demographics	4
Maraolo et al,^17^ 2017	Europe	Trainee association -microbiology	Cross-sectional	416	38	32	Microbiology	Residents	1, Own burnout questionnaire	Demographics, poor work environment	4
Ndom and Makanjuola,^56^ 2004	Nigeria	Teaching hospital	Cross-sectional	84	91	33	Multiple	Residents	2, List of stressors	Work demands, poor work environment, personal and self-efficacy, perceived/reported poor mental or physical health, demographics	2
Ochsmann et al^57^ 2011	Germany	Hospital	Cross-sectional	792	44	28.9	Multiple	All training grades	3, Recovery Stress Questionnaire	Work demands, poor work environment	7
Ogundipe et al,^58^ 2014	Nigeria	Hospital setting	Cross-sectional	204	58	33.4	Multiple	Residents	1,3, General Health Questionnaire and Maslach Burnout Inventory	Work demands, personal and self-efficacy, demographics, poor work environment	7
Ogunsemi et al,^12^ 2010	Nigeria	Hospital	Cross-sectional	58	74	35.7	Multiple	Residents	2,3, Measured perception and sources of stress and perception of well-being		3
										Demographics, poor career development, personal and self-efficacy, poor work environment, concerns about patient care, work demands, financial worries, perceived/reported poor mental or physical health	
Okpozo et al,^59^ 2017	US	3 Teaching hospitals	Cross-sectional	203	52	Not stated	Multiple	Residents	1, Maslach Burnout Inventory	Personal and self-efficacy, poor work environment	4
Pan et al,^60^ 2017	Australia	Hospital	Cross-sectional	540	40	Not stated	Multiple	Postgraduate Year 1-3	2, Perception of stress	Work demands, poor career development, personal and self-efficacy, poor work environment, concerns about patient care	8
Prins et al,^61^ 2010	Holland	Hospital	Cross-sectional	2115	39	31.5	Multiple	Residents	1, Maslach Burnout Inventory	Demographics, poor work-life balance, specialty	8
Saini et al,^62^ 2010	India	Hospital	Cross-sectional	721	53	27.5	Multiple	Residents	2,3, Depression Anxiety Stress Scale	Seniority, specialty, work demands, poor career development, personal and self-efficacy	5
Sochos et al,^63^ 2012	UK	Hospital and primary care	Cross-sectional	184	40	30.6	Multiple	All training grades	1, Maslach Burnout Inventory	Personal and self-efficacy, poor work environment, perceived/reported poor mental or physical health	4
Stucky et al,^4^ 2009	US	Hospital	Prospective study	144	36	Interns, 27.9; residents, 29.4	Pediatric and internal medicine	Interns and residents	3, Measuring emotional state every 90 min throughout each duty shift	Seniority, demographics, work demands, poor health	5
Taylor-East et al,^64^ 2013	Malta	Hospital	Cross-sectional	117	53	Not stated	Multiple	Foundation years 1 and 2	3, General Health Questionnaire	Seniority, demographics, personal and self-efficacy, poor career development	7
Toral-Villanueva et al,^65^ 2009	Mexico	Hospital	Cross-sectional survey	312	57	28	Multiple	All training grades	1, Maslach Burnout Inventory	Concerns about patient care, perceived/reported poor mental or physical health, work demands, seniority	8
Tyssen et al,^66^2005	Norway	Hospital	Longitudinal	371	58	29	Multiple	Interns	2, Modified version of Cooper Job Stress Questionnaire	Demographics, personal and self-efficacy, work demands, poor work environment	7
Verweij et al,^67^ 2017	Holland	Hospital	Cross-sectional	2115	39	31.5	Multiple	Residents	1, Maslach Burnout Inventory	Work demands, personal and self-efficacy, demographics, poor career development	8
West et al,^68^ 2011	US	Hospital	Cross-sectional	16394	55.7	Not stated	Multiple	Residents	1, Maslach Burnout Inventory	Demographics, financial worries, seniority	8
Woodside et al,^69^ 2008	US	Hospital and primary care	Cross-sectional	155	57	35	Family medicine and psychiatry	Residents	1, Maslach Burnout Inventory	Demographics, poor work-life balance, specialty	4
Zis et al,^70^2015	Greece	Hospital	Cross-sectional	116	45	34.5	Neurology	Residents	1, Maslach Burnout Inventory	Demographics, work demands, poor work-life balance, poor career development	7
Zubairi and Noordin,^71^ 2016	Pakistan	Hospital	Cross-sectional	110	54	Not stated	Multiple	Residents	1, Maslach Burnout Inventory	Work demands, poor work environment	4

^a^ Code for measure of wellness: 1, burnout (eg, Maslach Burnout Inventory); 2, stress (eg, Kessler-10 psychological distress instrument); and 3, other measures (eg, General Health Questionnaire).

**Table 2.** Factors Associated With Stress or Burnout Identified in This Review and Meta-analysis

Factors associated with burnout/stress	Description of outcomes	No. of studies
**Work-related**		
Poor work-life balance	Balance and potential interference between personal and professional life, including leisure time, family responsibilities, and influence of work on personal life	23
Concerns about patient care	Concerns around mistakes, poor patient outcomes, and suboptimal practices	9
Work demands	The work duties of trainee physicians, including workload, inefficient tasks, responsibility, job satisfaction, and on-call commitments	25
Seniority and grade	Level of training	11
Poor career development	Training opportunities, professional development, and job security	13
Specialties	Obstetrics and gynecology, pediatrics, medicine, surgery, psychiatry, and emergency	10
Poor work environment	Relationships at work, supervision and support, lack of feedback, negative work environment, size of residency program, and organizational constraints	19
**Non-work related**		
Financial worries	Perceived poor salary and financial problems and debt	8
Demographics	Sex, age, cultural background (eg, English as first language, migration, ethnicity, parental relationships)	16
		10
		7
Perceived/reported mental or physical poor health	Medical history, including mental health, nutrition, sleep, and lifestyle factors	8
Personal and self-efficacy	Control, autonomy, confidence, and self-efficacy	11

**Figure 2.**
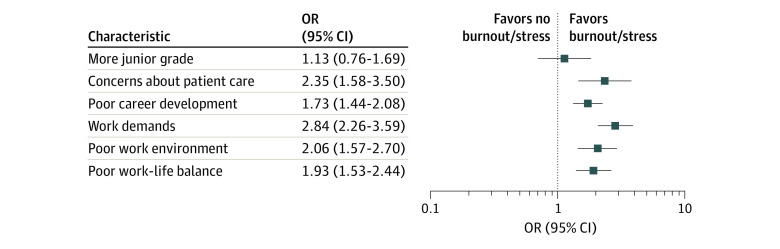
Meta-analysis of Each Work-Related Factor and Its Association With Burnout/Stress Each line represents 1 factor. Weights are from random-effects model. OR indicates odds ratio.

As shown in Figure 2, workplace-related demands were associated with nearly 3-fold increased odds for burnout/stress (OR, 2.84; 95% CI, 2.26-3.59; *I*^2^ = 88.8%; *P* < .001), followed by concerns about patient care (OR, 2.35; 95% CI, 1.58-3.50; *I*^2^ = 83.2%; *P* < .001), poor work environment (OR, 2.06; 95% CI, 1.57-2.70; *I*^2^ = 82.8%; *P* < .010), poor work-life balance (OR, 1.93; 95% CI, 1.53-2.44; *I*^2^ = 85.7%; *P* < .001), and poor career development (OR, 1.73; 95% CI, 1.44-2.08; *I*^2^ = 71.4%; *P* < .001). Forest plots of individual workplace-related and non–workplace-related factors can be found in eFigure 1 in the Supplement.

There was no association between higher rates of burnout/stress and seniority within trainee physicians (OR, 1.13; 95% CI, 0.76-1.69; *I*^2^ = 87.7%; *P* < .001). Studies were based on a range of different specialties, but there was no standard comparator specialty among the included studies; therefore, evaluating associations between burnout and specialties was challenging. The pooled estimate across 4 studies^31,56,61,69^ indicated that psychiatry was associated with a statistically significant higher level of burnout/stress (OR, 1.41; 95% CI, 1.1-1.8; *I*^2^ = 22.8%; *P* = .27) compared with family medicine and surgery (eFigure 2 in the Supplement).

**Figure 3.**
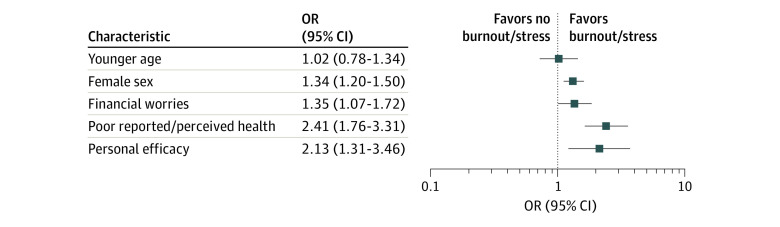
Meta-analysis of Each Non–Work-Related Factor and Its Association With Burnout/Stress Each line represents 1 factor. Weights are from random-effects model. OR indicates odds ratio.

Findings on non–work-related factors showed an association with increased odds for burnout/stress for perceived/reported poor mental or physical health (OR, 2.41; 95% CI, 1.76-3.31; *I*^2^ = 70.1%; *P* = .001), low personal and self-efficacy (OR, 2.13; 95% CI, 1.31-3.46; *I*^2^ = 93.6%; *P* < .001), financial worries (OR, 1.35; 95% CI, 1.07-1.72; *I*^2^ = 62.7%; *P* = .009), and female sex (OR, 1.34; 95% CI, 1.20-1.50; *I*^2^ = 41.7%; *P* = .05) (Figure 3). Younger age (OR, 1.02; 95% CI, 0.78-1.34; *I*^2^ = 59.6%; *P* = .008) was not associated with burnout/stress. Owing to the heterogeneous data for culture and background, which included measures such as migration,^64^ spoken language,^31^ upbringing,^43^ ethnicity,^34^ and whether trainees were accustomed to US culture,^69^ it was not possible to pool data; thus, the ORs of each study are presented in a forest plot (eFigure 1B in the Supplement).

Pooled ORs for most outcomes in the 3 sensitivity analyses (studies based only on burnout and Maslach Burnout Inventory measures; studies with ≥6 scorings on the adapted NOS) did not differ significantly from the pooled ORs reported in the main analyses (eTable 2 in the Supplement). However, no association was found with personal and self-efficacy when only burnout and Maslach Burnout Inventory measures were included.

The Egger test was undertaken for the pooled ORs of poor career development, female sex, more junior training level, concerns about patient care, work demands, poor work environment, and poor work-life balance. No evidence for publication bias was obtained for all pooled outcomes except work demands. The pooled OR between work demands and burnout/stress may be influenced by publication bias (regression intercept, 2.95; SE, 0.96; *P* = .006). Individual funnel plots can be found in eFigure 3 in the Supplement.

## Discussion

This systematic review and meta-analysis of 48 studies across 36 266 trainee physicians examined a range of factors associated with burnout and stress. The reviewed evidence suggests that trainee physicians reporting negative workplace conditions, such as dysfunctional work environment, excessive work demands, and concerns about patient care, were 2 times more likely to report burnout/stress. We also found evidence that some non–work-related factors may be associated with burnout/stress in trainee physicians, but most of these appear to be less important than workplace factors and less robust based on our sensitivity analyses.

Two previous literature reviews have focused on burnout and explored the association between contributing factors and burnout in trainee physicians.^16,72^ These were not systematic reviews, but identified high work demands, poor work-life balance, poor control, and poor work environments as potential contributors.^15,41^

In our study, we undertook meta-analysis, enabling the quantifications and comparisons of these links and allowing for exploration of key sources of heterogeneity among the studies. We chose to focus on all trainee physicians engaged in postgraduate training to understand the factors associated with burnout/stress in trainee physicians as a group, as previous reviews have focused on residents. Including physicians only at residency grade may not take into account different nomenclature used in different countries, as well as other trainee physician grades (eg, interns).

Control and personality have been implicated in previous literature reviews,^16,72^ and our study found an association between personal and self-efficacy and burnout/stress in the overall analyses. However, this association was less robust after sensitivity analyses were performed. This weaker association could be due to differences in assessments, but these factors could also be affected by support and coping, which could moderate the association.^72^ Further high-quality studies are required to explore this factor in more detail.

Our results support the need for organizational interventions, which is in line with previous reviews.^73^ Most studies that evaluated interventions to reduce burnout have focused on physician-directed interventions, such as mindfulness and building self-confidence.^73^ Studies that have tested organizational interventions tend to focus mostly on modifying shift patterns and workload,^73^ but few studies have incorporated interventions that try to address multiple organizational factors, including improved teamwork, workflow, and organizational restructuring,^73,74,75^ which may be more useful in reducing burnout. Our findings suggest a need to shift to research agendas that target the organizational environment, improving working relationships among physicians and other health care professionals, as well as promoting work-life balance to mitigate burnout in trainee physicians. Although organizational interventions are generally considered costly and time-consuming, they may still be efficient and cost-effective owing to increased retention of physicians and improved quality of patient care.^76,77^

Among specialties, psychiatry was found to be associated with particularly high risk for burnout/stress. There might also be additional high-risk specialties that we could not detect owing to the high heterogeneity and lack of consistency in the comparator groups (surgery, internal medicine, family medicine, psychiatry, and emergency medicine) used across studies. Moreover, burnout is prevalent across all specialties, which makes it difficult to identify significant differences at the specialty level.^5,14^ Burnout symptoms have been found to differ among different specialties, which could indicate that there are some systematic differences in working conditions that are associated with burnout between different specialties.^15^ In obstetrics and gynecology, high litigation levels and workforce retention^78^ have been factors associated with burnout; however, only 3 studies in our review investigated this specialty. Regarding psychiatry, it has been suggested that over one-third of psychiatry trainees met the criteria for severe burnout, and reasons for leaving included job stress, unsuitability, and concerns about lack of evidence-based treatments.^79,80^

Female trainee physicians showed an association with burnout/stress that is consistent with previous research.^15^ This association could be due to higher work-life interference, especially among women with younger children.^15,81^ Moreover, there have been reports that workplace sexual harassment and sex-based discrimination can contribute to burnout.^82^ Based on our findings, further research is warranted to develop appropriate interventions to mitigate burnout/stress in these higher-risk groups (eg, women and psychiatry trainees).

We found that workplace-related factors, such as poor work environment, excessive work demands, and poor work-life balance, were statistically significantly associated with burnout/stress. Poor work-life balance has been found to affect physicians in general^15^ (ie, not just trainee physicians), but other contributing factors present during training, such as postgraduate training requirements conflicting with personal life, could further affect work-life balance. One aspect of work environment mentioned by Prins et al^83^ was support and satisfying work relationships. Lack of senior support and feedback have been associated with physician burnout, whereas residents with mutually beneficial supervision were found to have lower levels of burnout,^84^ which suggests that support may have a buffering effect on burnout.^85^ In addition to the supervisor and trainee physician relationship, coherent team structures may also protect against burnout.^86^ It is likely that workplace-related and non–workplace-related factors interact and dynamically influence each other, which in turn suggests the need for multicomponent interventions focusing on individuals as well as organizations.

### Strengths and Limitations

To our knowledge, this is the first systematic review and meta-analysis exploring factors associated with burnout/stress in trainee physicians. Undertaking meta-analysis enabled comparisons to be made between workplace- and non–workplace-related factors associated with burnout/stress and examination of the consistencies of the associations. In addition, this review was performed and reported according to the PRISMA and MOOSE guidelines.^18,19^

There are limitations to the study. A wide range of factors associated with burnout/stress were included in this review, some of which had to be pooled in the same category (eg, work demands). We accounted for large heterogeneity by applying random-effects models to adjust for study-level variations. Another possible solution could be to apply subgroup and meta-regression analyses, but such analyses are not advisable when the pooled associations are based on a relatively small number of studies (eg, <20/outcome). Furthermore, owing to the intrinsic limitation of the study design, it is not possible to identify a specific joint model to investigate combined contributions across factors. We suggest that future empirical studies be conducted to examine the joint contribution of the core factors that we found to be associated with burnout.

An eligibility criterion to ensure feasibility of this review was that studies explicitly stated that they examined factors associated with burnout/stress. Although we searched multiple bibliographic databases and screened the references of the eligible studies, studies that did not state that factors associated with burnout/stress (eg, in titles, abstracts, or key words) were investigated may have not been captured by our searches. We excluded gray literature because unpublished studies are generally of lower quality and are more difficult to combine than peer-reviewed articles.^87^ We also excluded non-English language articles, although our search did not identify any eligible studies excluded solely based on language.

It could be argued that meta-analysis is inappropriate in the context of high levels of method and statistical heterogeneity and it may have been more appropriate to summarize the results as a narrative review. However, meta-analysis enabled us to compare results across studies, examine the consistency of associations, and present the results in a way that facilitates interpretation compared with lengthy narratives.^88^ In addition, most of the studies included in our review were cross-sectional and hence we are not able to establish direct links between contributing factors and burnout/stress. Large, prospective investigations are needed to rigorously examine contributors to burnout/stress in trainee physicians over time.

## Conclusions

The findings of this study suggest that burnout/stress in trainee physicians is predominantly associated with workplace-related factors, such as work demands and poor work environment, rather than nonmodifiable and non–workplace-related factors. Multilevel organizational interventions targeting poor work environment and work demands have the potential to mitigate burnout and stress in trainee physicians.

## Notes

Supplement. **eTable 1.** Search Strategy **eTable 2.** Comparison of Pooled Outcome Sizes of Main Analyses and Sensitivity Analyses **eFigure 1.** Forest Plots of Association Between Burnout and Different Factors **eFigure 2.** Meta-analysis of Each Individual Specialty and Its Association With Burnout/Stress **eFigure 3.** Funnel Plots **eMethods.** Systematic Review Protocol **eReferences**
