# Supplementary material for: Factors Associated With Burnout and Stress in Trainee Physicians: A Systematic Review and Meta-analysis
Source: JAMA Netw Open. 2020 Aug 18;3(8):e2013761. doi: 10.1001/jamanetworkopen.2020.13761 (PMC7435345; doi:10.1001/jamanetworkopen.2020.13761)
Supplement: Supplement. — eTable 1. Search Strategy eTable 2. Comparison of Pooled Outcome Sizes of Main Analyses and Sensitivity Analyses eFigure 1. Forest Plots of Association Between Burnout and Different Factors eFigure 2. Meta-analysis of Each Individual Specialty and Its Association With Burnout/Stress eFigure 3. Funnel Plots eMethods. Systematic Review Protocol eReferences [file jamanetwopen-3-e2013761-s001.pdf]

Supplementary Online Content

Zhou AY, Panagioti M, Esmail A, Agius R, Van Tongeren M, Bower P. Factors associated with burnout and stress in trainee physicians: a systematic review and meta-analysis. *JAMA Netw Open*. 2020;3(8):e2013761. doi:10.1001/jamanetworkopen.2020.13761

eTable 1. Search Strategy

eTable 2. Comparison of Pooled Outcome Sizes of Main Analyses and Sensitivity Analyses

eFigure 1. Forest Plots of Association Between Burnout and Different Factors

eFigure 2. Meta-analysis of Each Individual Specialty and Its Association With Burnout/Stress

eFigure 3. Funnel Plots

eMethods. Systematic Review Protocol

eReferences

This supplementary material has been provided by the authors to give readers additional information about their work.

eTable 1. Search strategy

1	distress*
2	nervous*
3	Stress, Psychological/ or stress*
4	Anxiety/ or anxiety
5	apprehension
6	worry*
7	Burnout, Professional/ or burnout*
8	or/1-7
9	((trainee or trainee or foundation year or registrar or resident) adj2 (physician* or doctor*))
10	(speciality trainee* or intern)
11	Physicians/
12	(trainee or trainee or foundation year or registrar)
13	11 and 12
14	9 or 10 or 13
15	(determinant* or factor* or driver* or caus* or contributor* or stressor* or predictor* or predispos* or correlat* or associat* or risk*)
16	8 and 14 and 15

© 2020 Zhou AY et al. *JAMA Network Open.*

**eTable 2.** comparison of pooled outcome sizes of main analyses and sensitivity analyses								
**Category**	**Main analyses OR (95% C.I)**	**Heterogeneity**	**Sensitivity analysis burnout only OR (95% C.I)**	**Heterogeneity**	**Sensitivity analysis Maslach Burnout Inventory only OR (95% C.I)**	**Heterogeneity**	**Sensitivity analysis Newcastle Ottawa score≥6 only OR (95%C.I)**	**Heterogeneity**
**Younger age**	1.02 (0.78,1.34)	59.6%, p=0.008	1.18 (0.79,1.75)	69.2%, p<0.006	n/a	n/a	0.93 (0.63, 1.37)	64.1%, p=0.025
**Female gender**	1.34 (1.20, 1.50)	41.7%, p=0.045	1.27 (1.19, 1.36)	9.4%, p=0.36	1.25 (1.18,1.32)	0%, p=0.63	1.25(1.19, 1.32)	0%, p=0.54
**Financial worries**	1.35 (1.07, 1.72)	62.7%, p=0.009	n/a	n/a	n/a	n/a	n/a	n/a
**Reported/ perceived poor mental or physical health**	2.41 (1.76, 3.31)	70.1%, p=0.001	1.96 (1.36, 2.84)	36.4%, p=0.18	n/a	n/a	2.02 (1.68, 2.44)	10.6%, p=0.35
**More Junior grade**	1.13 (0.76, 1.69)	87.7%, p<0.001	0.93 (0.63, 1.35)	69.1%, p=0.004	0.85 (0.48,1.51)	79.0%, p=0.001	0.79 (0.50, 1.25)	77.1%, p=0.001
**Concerns about Patient care**	2.35 (1.58, 3.50)	83.2% p<0.001	n/a	n/a	n/a	n/a	2.7 (1.58, 4.62)	89.4%, p=0.000
**Poor Personal efficacy**	2.13 (1.31, 3.46)	93.6%, p<0.001	1.52 (0.77, 3.03)	91.1%, p<0.001	1.33 (0.65,2.75)	91.7%, p<0.001	n/a	n/a
**Poor career development**	1.73 (1.44, 2.08)	71.4%, p<0.001	n/a	n/a	n/a	n/a	1.78 (1.38, 2.29)	65.7%, p=0.008
**Work demands**	2.84 (2.26, 3.59)	88.8% p<0.001	2.92 (1.71, 4.98)	88.4%, p<0.001	2.30 (1.53,3.45)	73.0%, p=0.001	2.57 (1.86, 3.56)	90.7%, p<0.001
**Poor Work environment**	2.06 (1.57, 2.7)	82.8%, p<0.001	1.94 (1.38, 2.73)	70.2%, p<0.001	2.21 (1.47,3.33)	69.7%, p=0.002	1.57 (1.34, 1.84)	5.3%, p=0.39
**Poor Work-life balance**	1.93 (1.53, 2.44)	85.7%, p<0.001	1.46 (1.08, 1.97)	85.4%, p<0.001	1.61 (1.15,2.25)	87.0%, p<0.001	1.87 (1.27, 2.74)	87.5%, p<0.001
**Culture and upbringing**	no overall	n/a	n/a	n/a	n/a	n/a	n/a	n/a
**Emergency medicine**	1.63 (0.48, 5.53)	82.3%, p=0.004	n/a	n/a	n/a	n/a	n/a	n/a
**Obstetrics and gynaecology**	2.12 (0.8, 5.64)	48.7%, p=0.14	n/a	n/a	n/a	n/a	n/a	n/a

© 2020 Zhou AY et al. *JAMA Network Open.*

**Internal Medicine**	1.2 (0.67, 2.14)	73.5%, p=0.002	1.20 (0.67, 2.14)	73.5%, p=0.002	n/a	n/a	n/a	n/a
**Paediatrics**	1.07 (0.7, 1.65)	16.5%, p= 0.30	n/a	n/a	n/a	n/a	n/a	n/a
**Psychiatry**	1.41 (1.1, 1.8)	22.8%, p=0.27	n/a	n/a	n/a	n/a	n/a	n/a
**Surgery**	1.46 (0.86, 2.49)	76.1%, p<0001	1.13 (0.77, 1.66)	53.7%, p=0.071	n/a	n/a	n/a	n/a

© 2020 Zhou AY et al. *JAMA Network Open.*

eFigure 1. forest plots of association between burnout and different factors

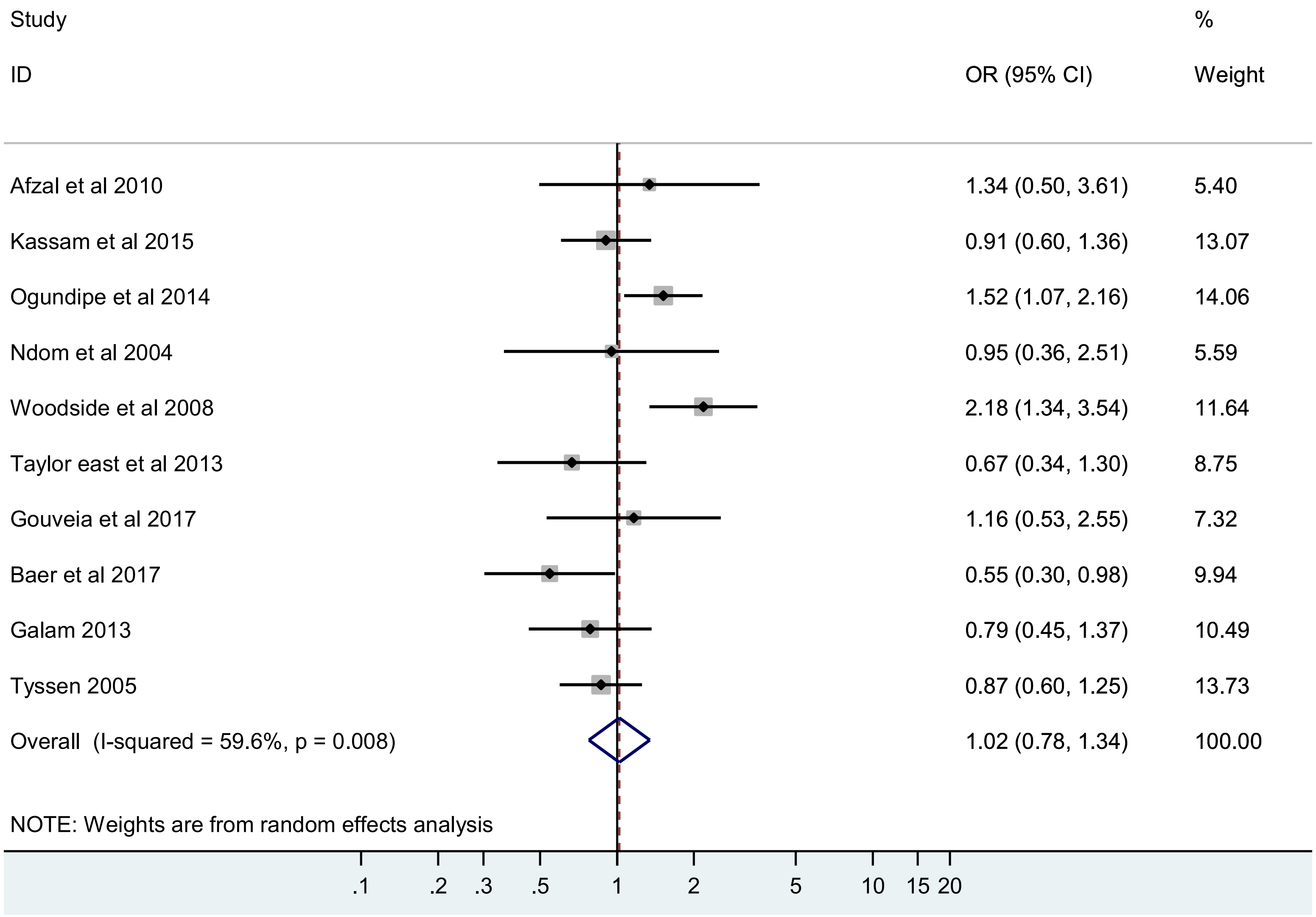

© 2020 Zhou AY et al. *JAMA Network Open.*

eFigure 1a Subgroup analysis of individual study and pooled effects of the association between younger age and burnout/distress. Each line represents 1 study in the meta-analysis. Weights are from random-effects model.

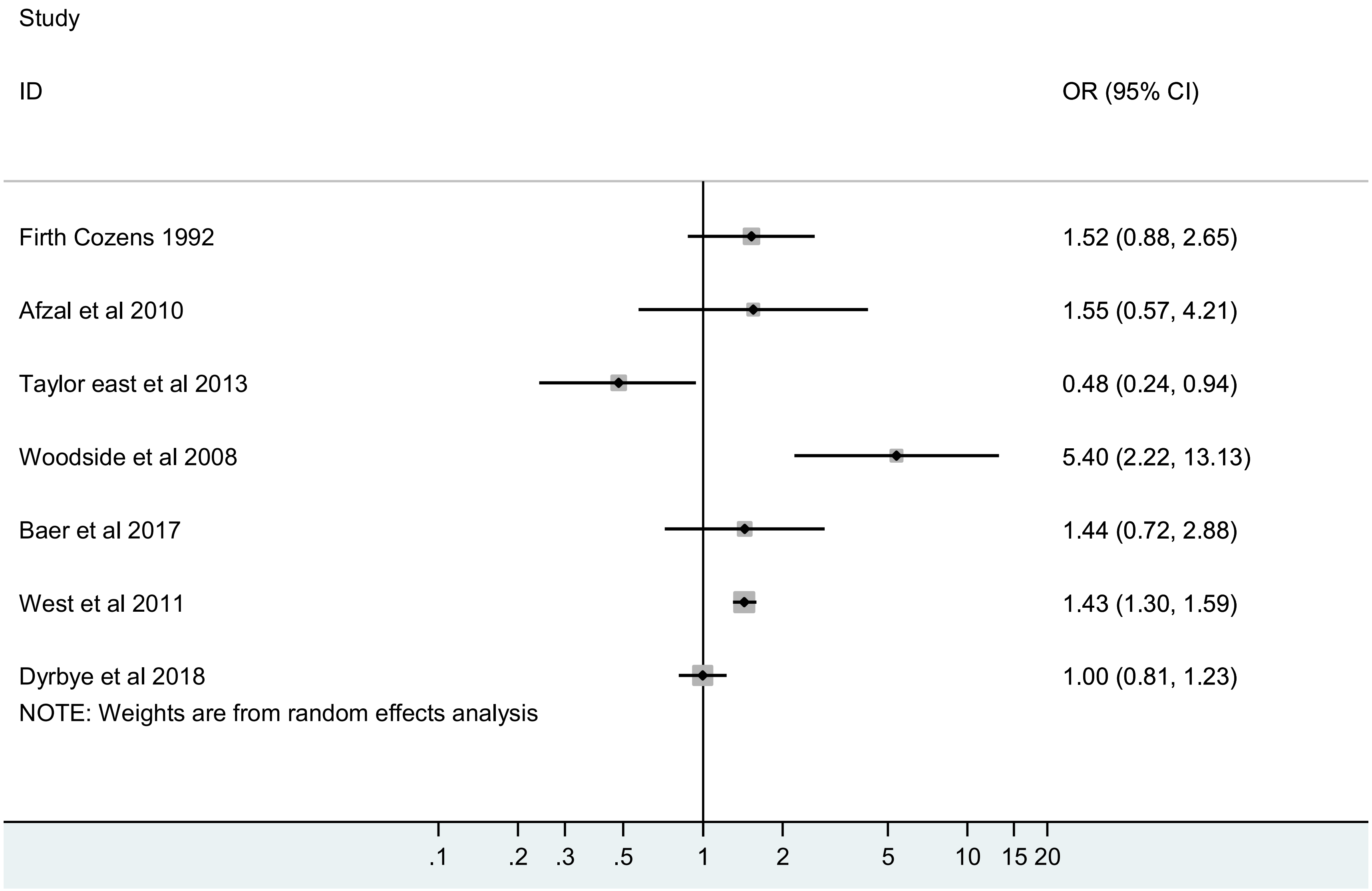

eFigure 1b Analysis of individual studies of the association between cultural and upbringing factors and burnout/distress. Each line represents 1 study in the meta-analysis. Weights are from random-effects model.

© 2020 Zhou AY et al. *JAMA Network Open.*

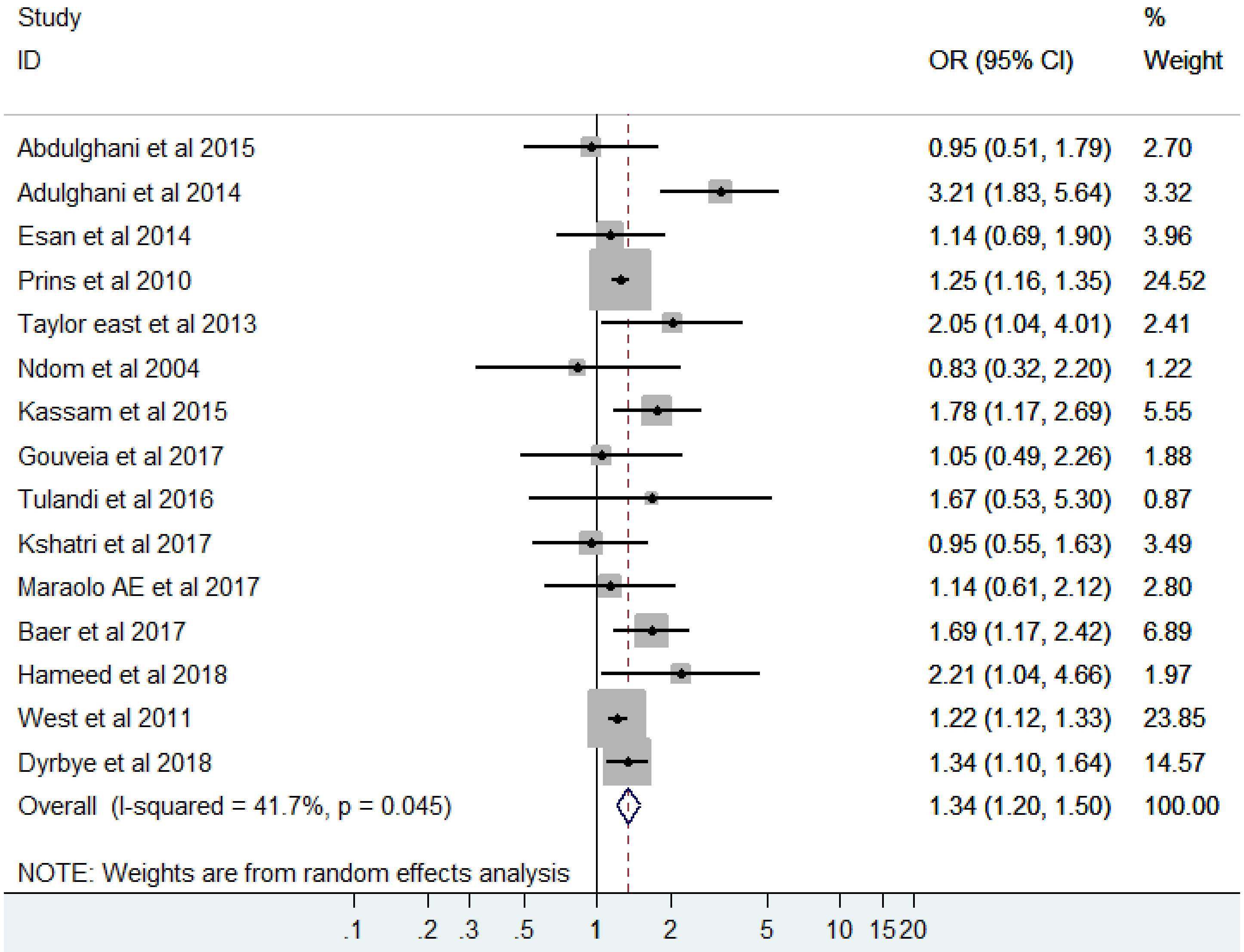

eFigure 1c Subgroup analysis of individual study and pooled effects of the association between female gender and burnout/distress. Each line represents 1 study in the meta-analysis. Weights are from random-effects model.

© 2020 Zhou AY et al. *JAMA Network Open.*

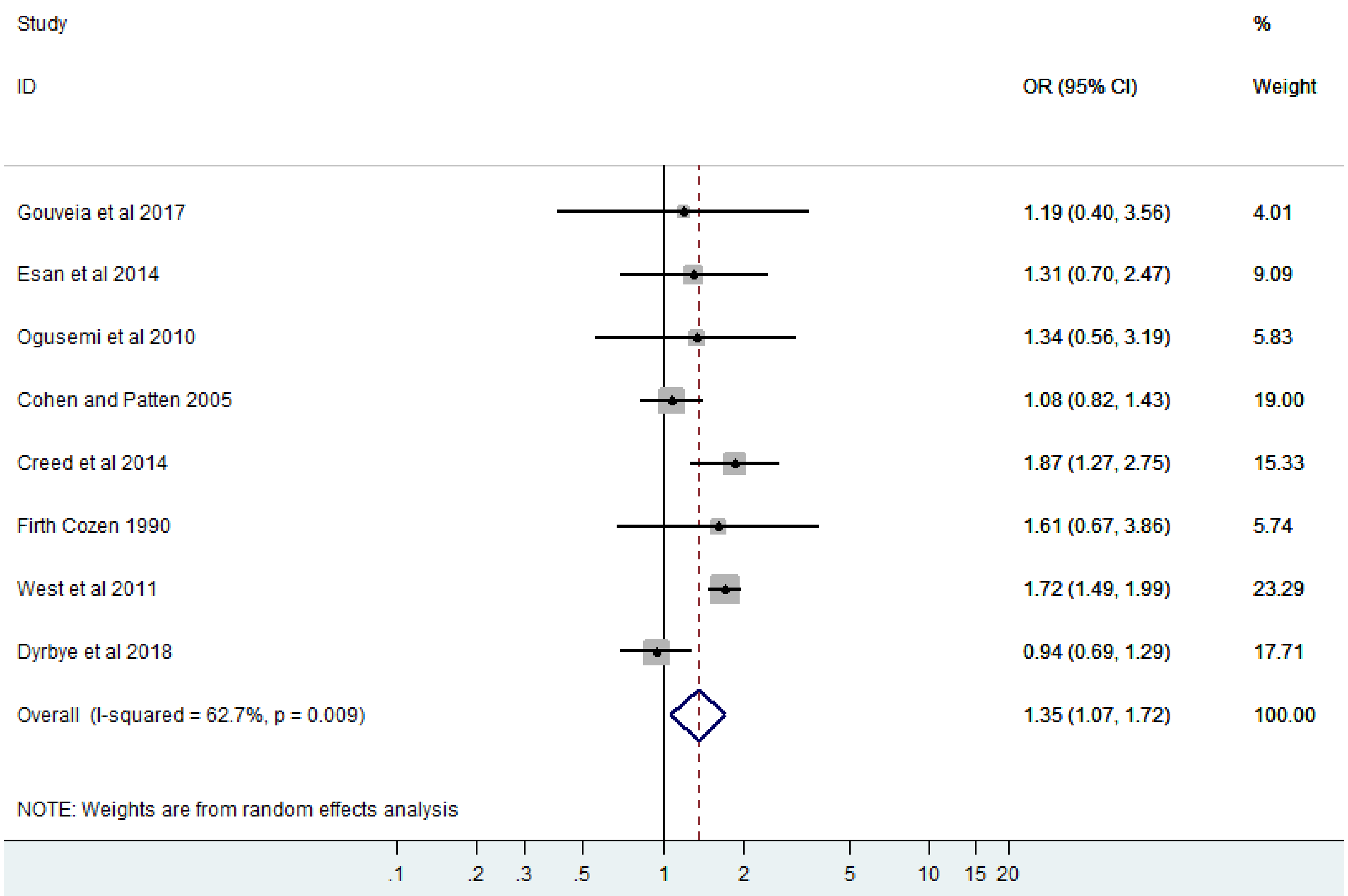

eFigure 1d Subgroup analysis of individual study and pooled effects of the association between financial worries and burnout/distress.Each line represents 1 study in the meta-analysis. Weights are from random-effects model.

© 2020 Zhou AY et al. *JAMA Network Open.*

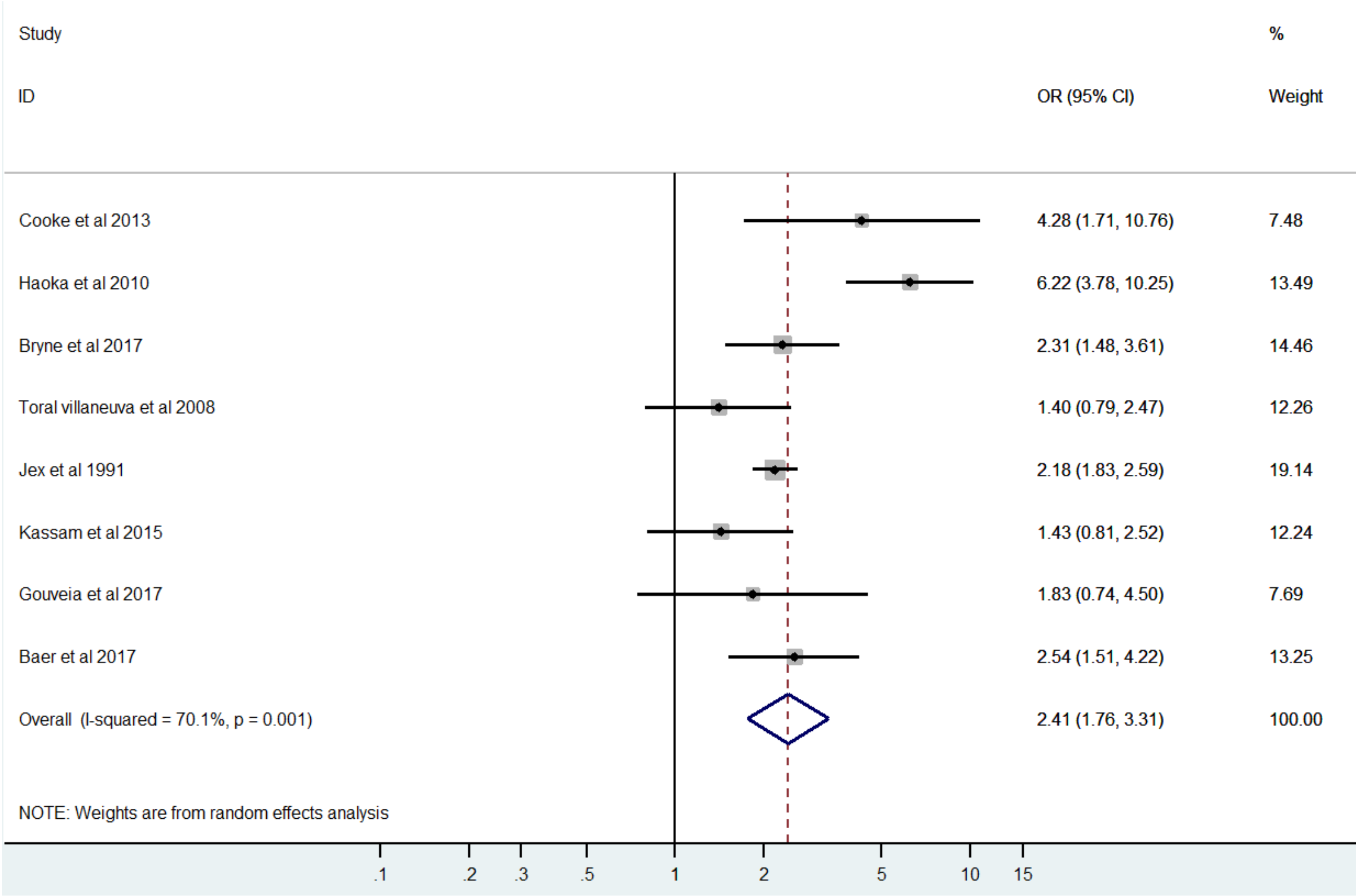

eFigure 1e Subgroup analysis of individual study and pooled effects of the association between reported/perceived poor mental or physical health and burnout/distress.Each line represents 1 study in the meta-analysis. Weights are from random-effects model.

© 2020 Zhou AY et al. *JAMA Network Open.*

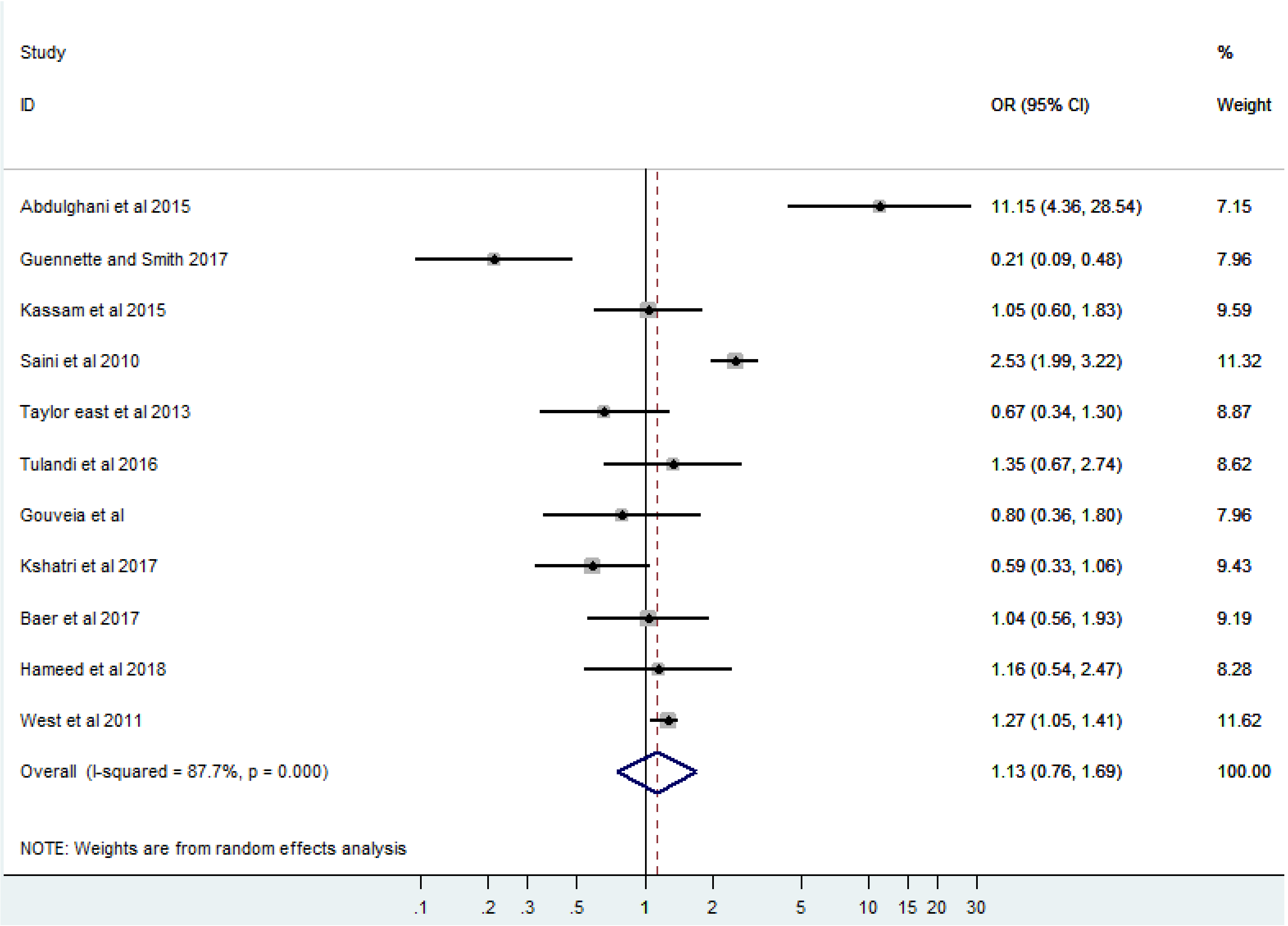

eFigure 1f Subgroup analysis of individual study and pooled effects of the association between more junior grade and burnout/distress.Each line represents 1 study in the meta-analysis. Weights are from random-effects model.

© 2020 Zhou AY et al. *JAMA Network Open.*

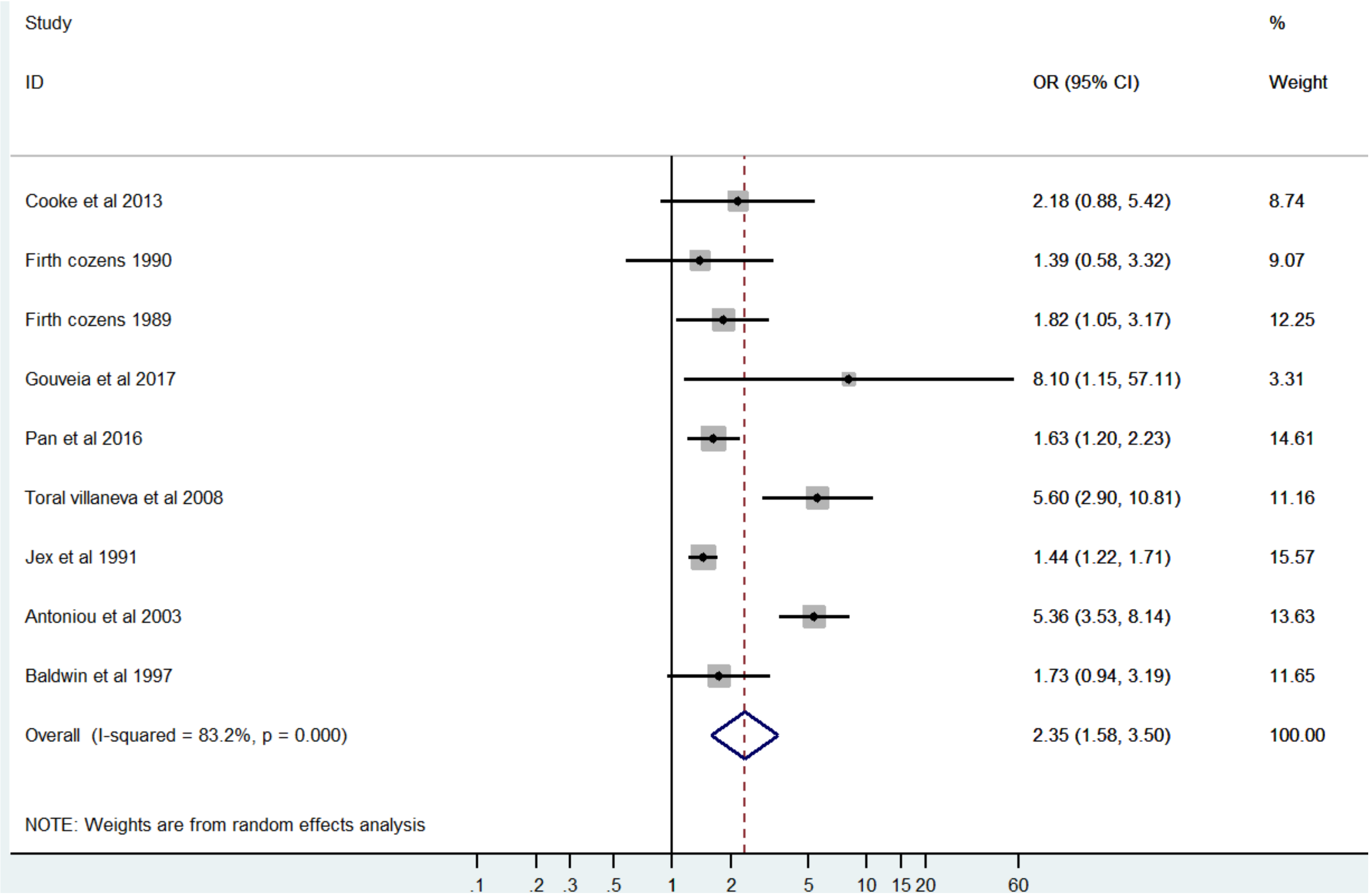

eFigure 1g Subgroup analysis of individual study and pooled effects of the association between concerns about patient care and burnout/distress.Each line represents 1 study in the meta-analysis. Weights are from random-effects model.

© 2020 Zhou AY et al. *JAMA Network Open.*

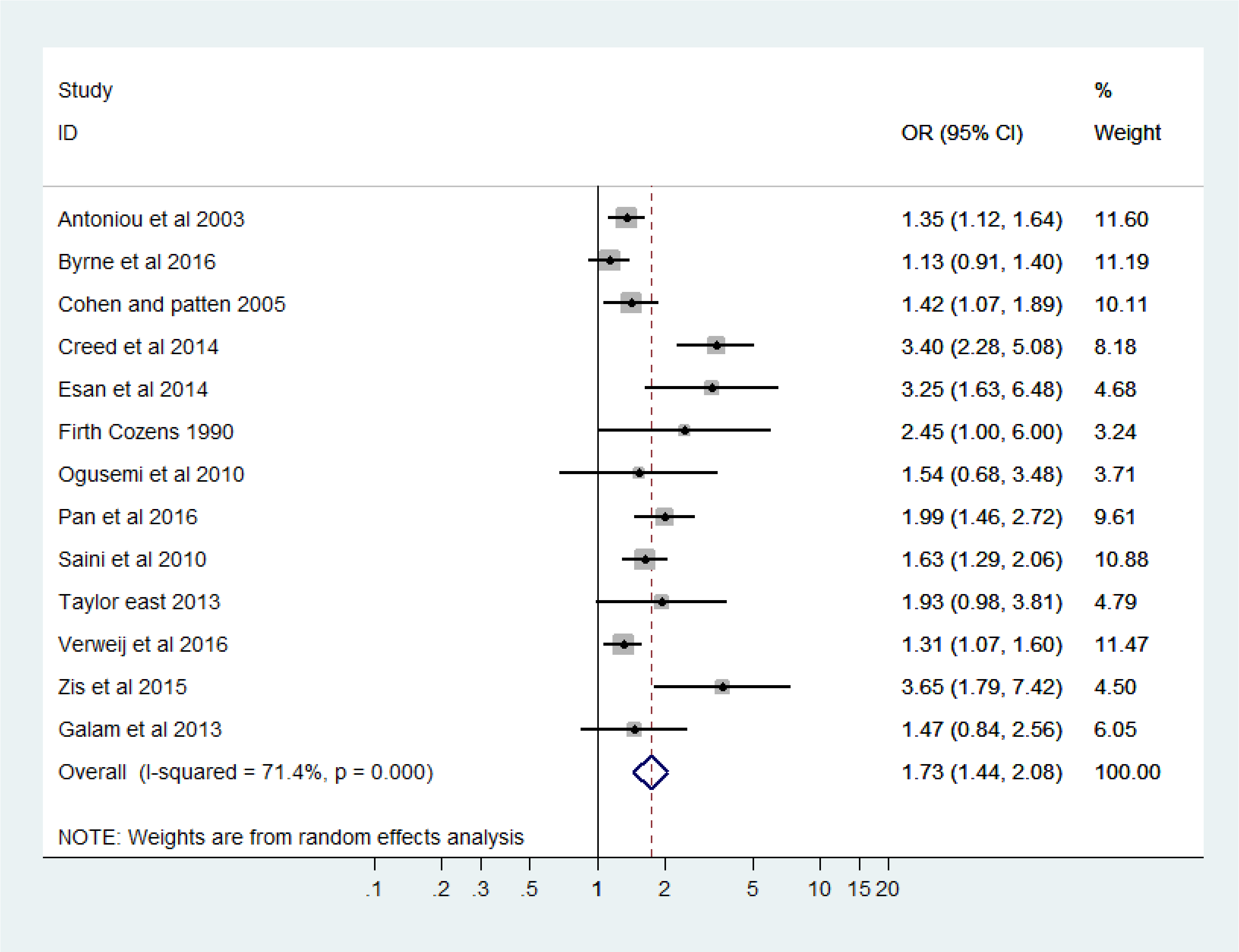

eFigure 1h Subgroup analysis of individual study and pooled effects of the association between poor career development and burnout/distress. Each line represents 1 study in the meta-analysis. Weights are from random-effects model.

© 2020 Zhou AY et al. *JAMA Network Open.*

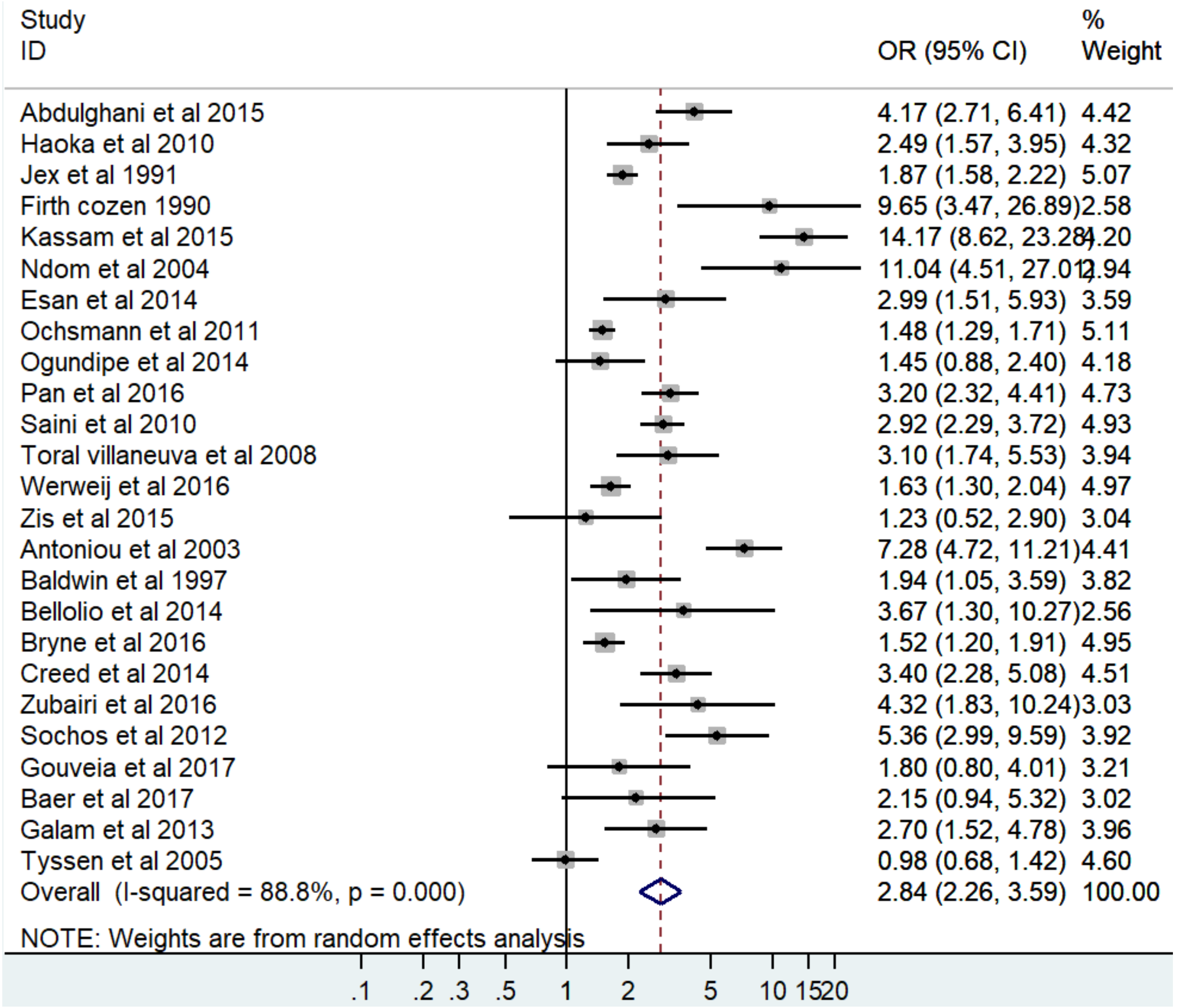

eFigure 1i Subgroup analysis of individual study and pooled effects of the association between work demands and burnout/distress. Each line represents 1 study in the meta-analysis. Weights are from random-effects model.

© 2020 Zhou AY et al. *JAMA Network Open.*

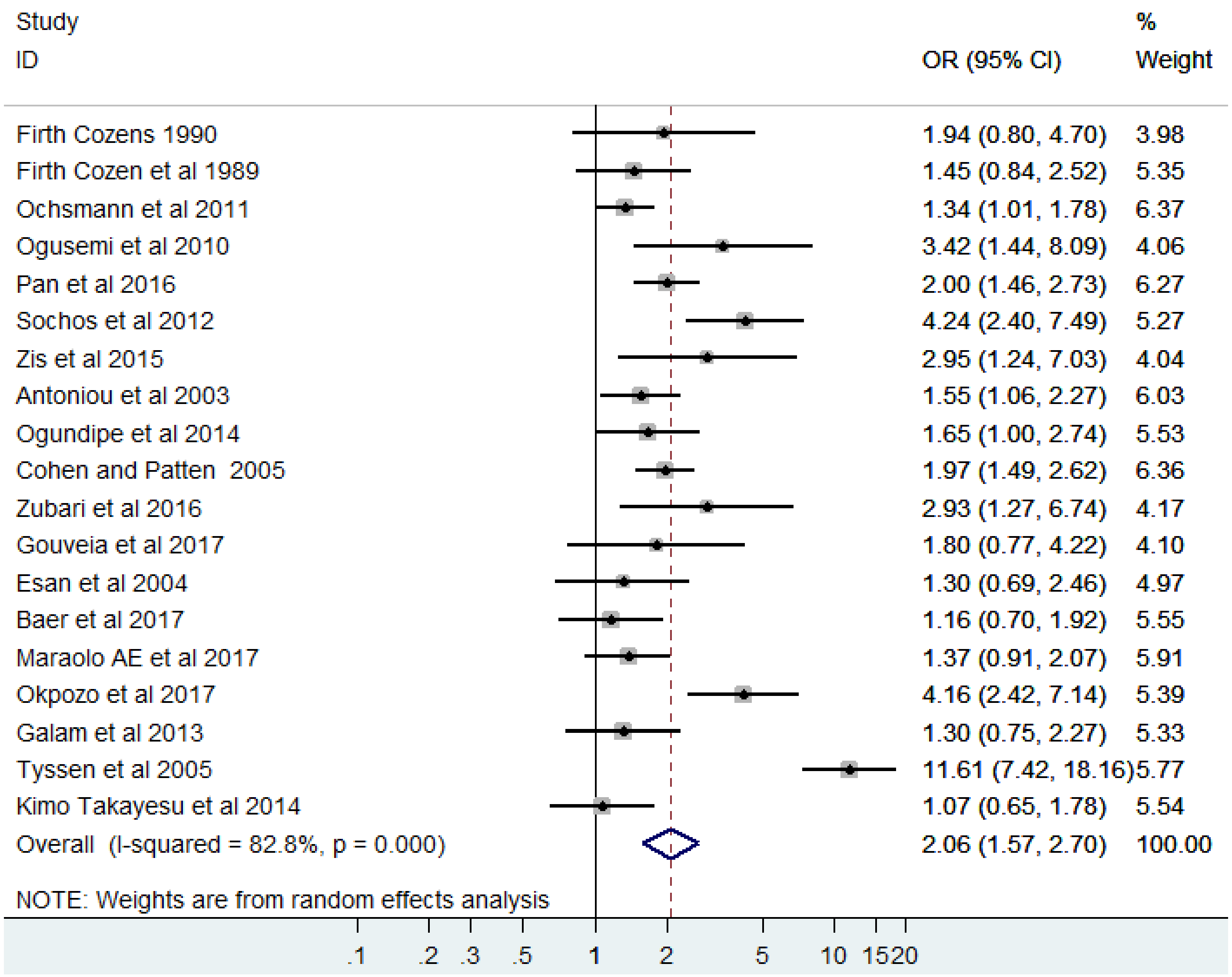

eFigure 1j Subgroup analysis of individual study and pooled effects of the association between work environment and burnout/distress.Each line represents 1 study in the meta-analysis. Weights are from random-effects model.

© 2020 Zhou AY et al. *JAMA Network Open.*

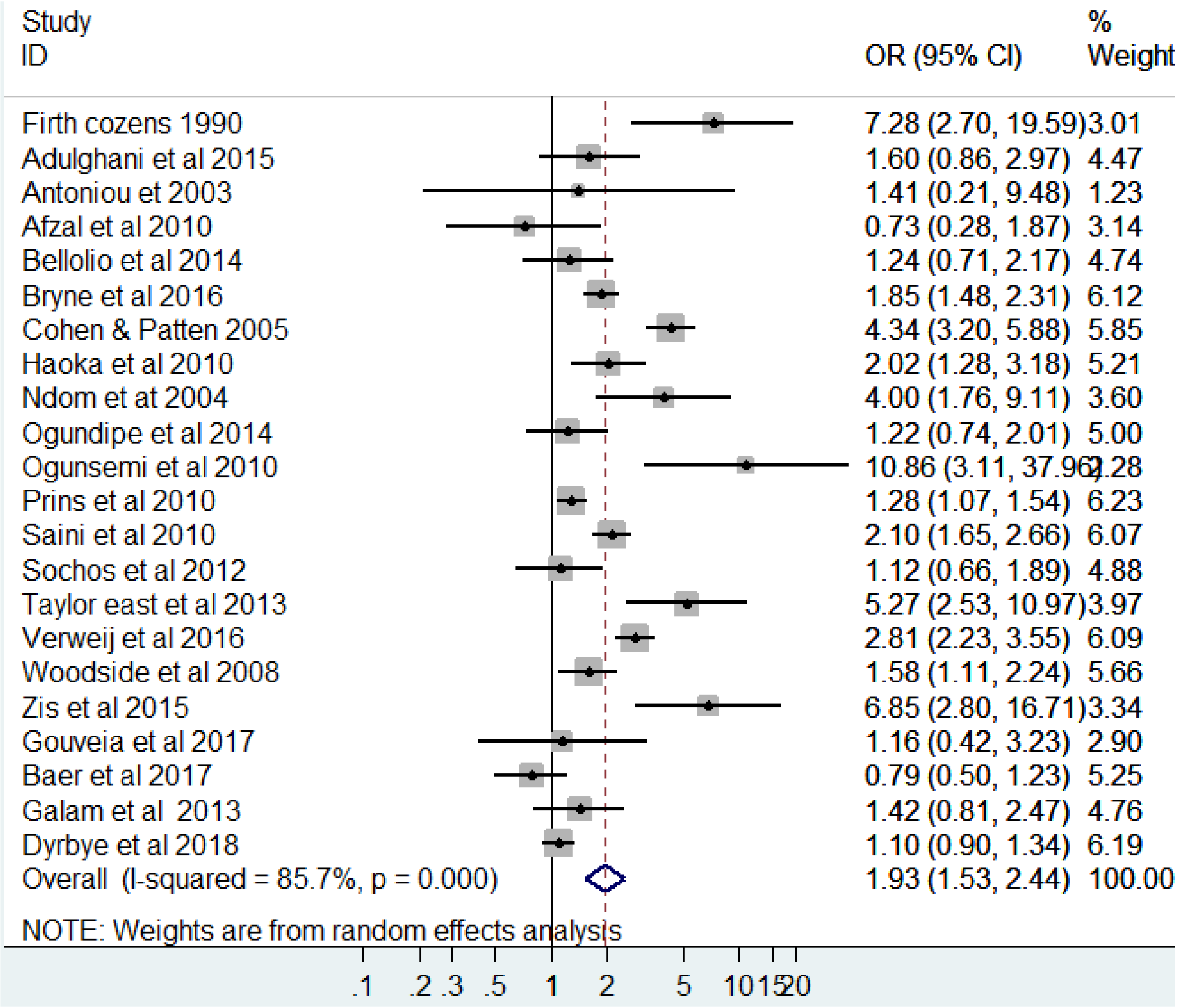

eFigure 1k Subgroup analysis of individual study and pooled effects of the association between poor work-life balance and burnout/distress.Each line represents 1 study in the meta-analysis. Weights are from random-effects model.

© 2020 Zhou AY et al. *JAMA Network Open.*

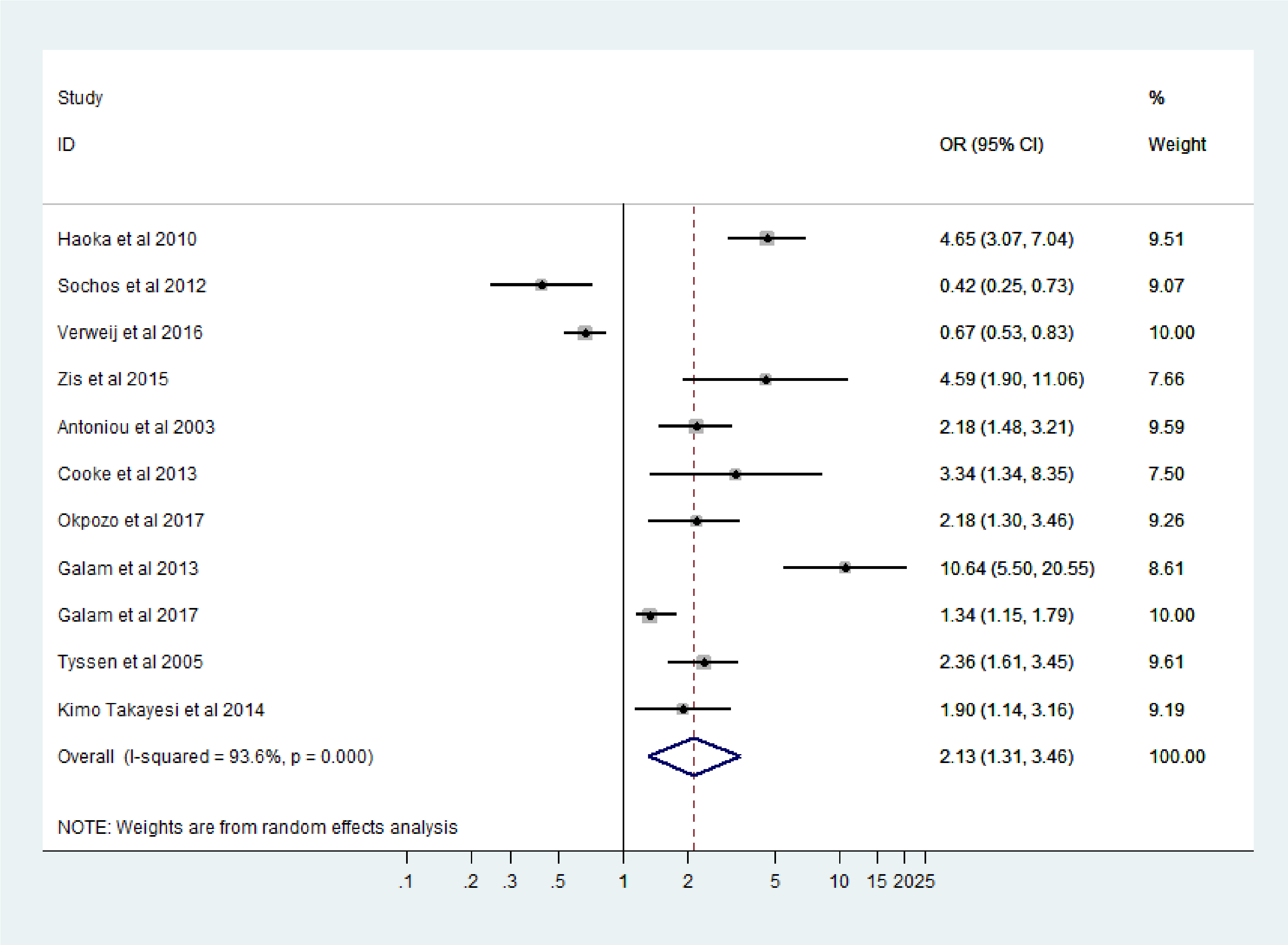

eFigure 1i Subgroup analysis of individual study and pooled effects of the association between Poor Personal efficacy and burnout/distress.Each line represents 1 study in the meta-analysis. Weights are from random-effects model.

© 2020 Zhou AY et al. *JAMA Network Open.*

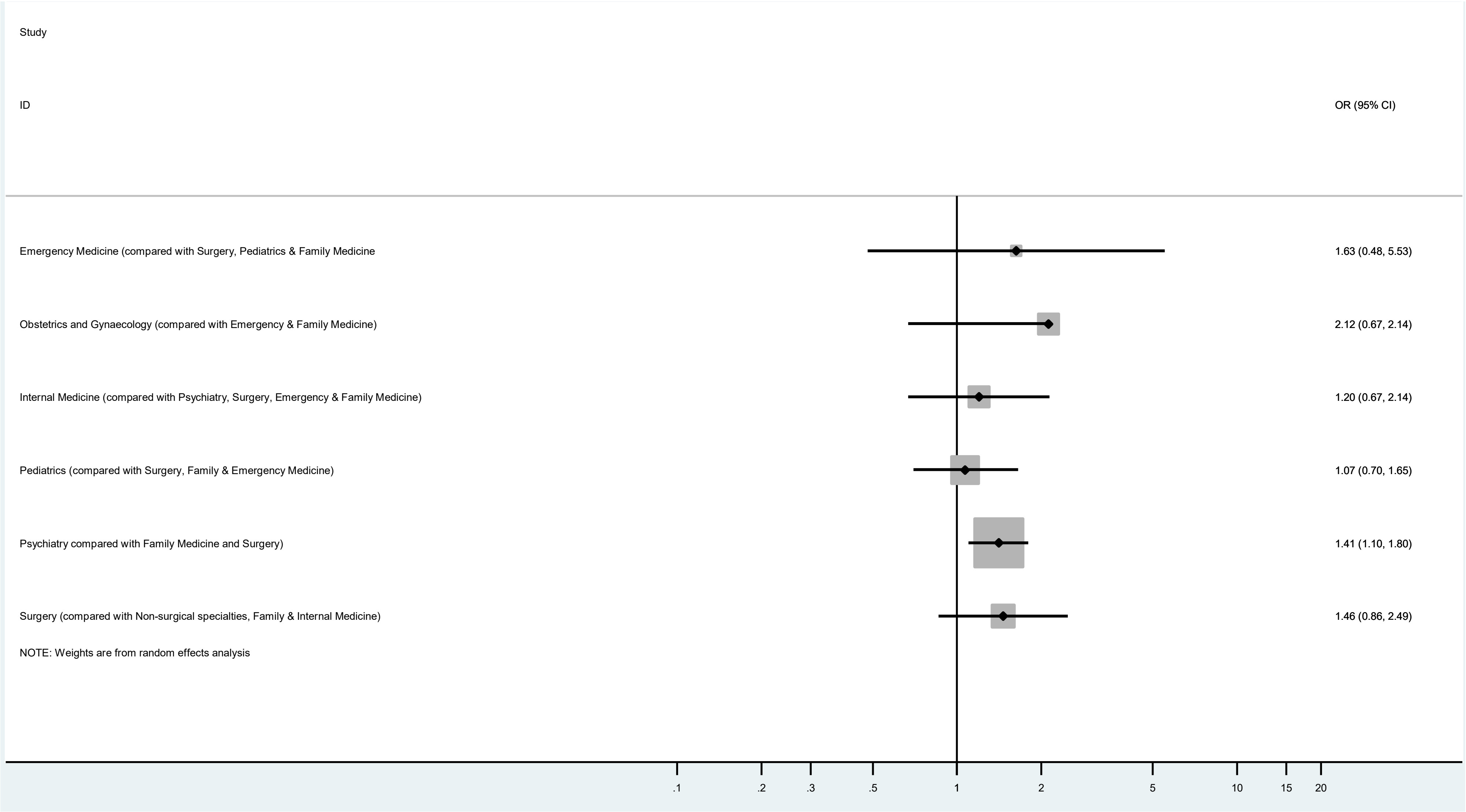

eFigure 2 Meta-analysis of each individual specialty and its association with burnout/stress. Each line represents 1 specialty. Weights are from random-effects model.

© 2020 Zhou AY et al. *JAMA Network Open.*

eFigure 3. Funnel plots

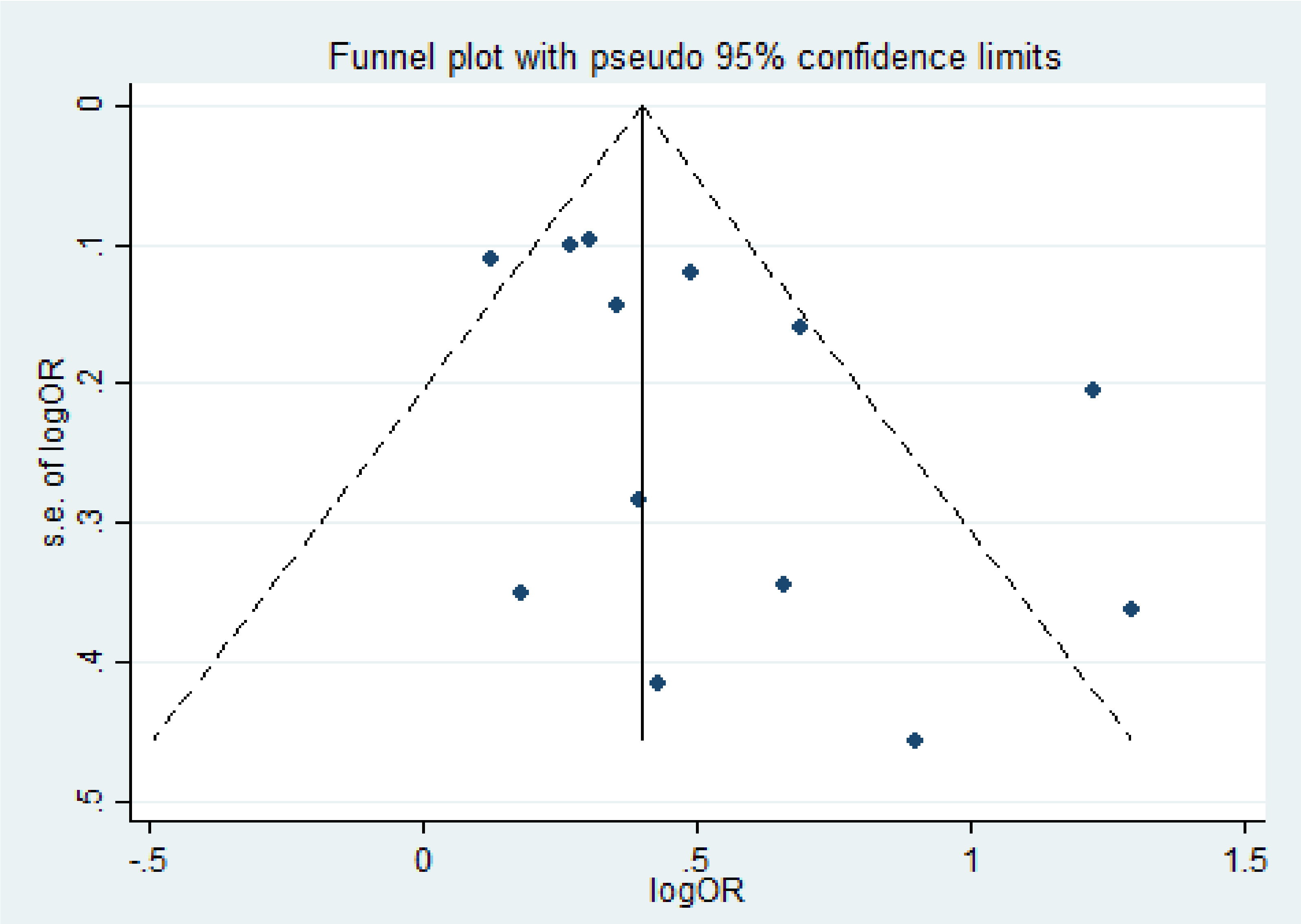

eFigure 3a Funnel Plot for career development

© 2020 Zhou AY et al. *JAMA Network Open.*

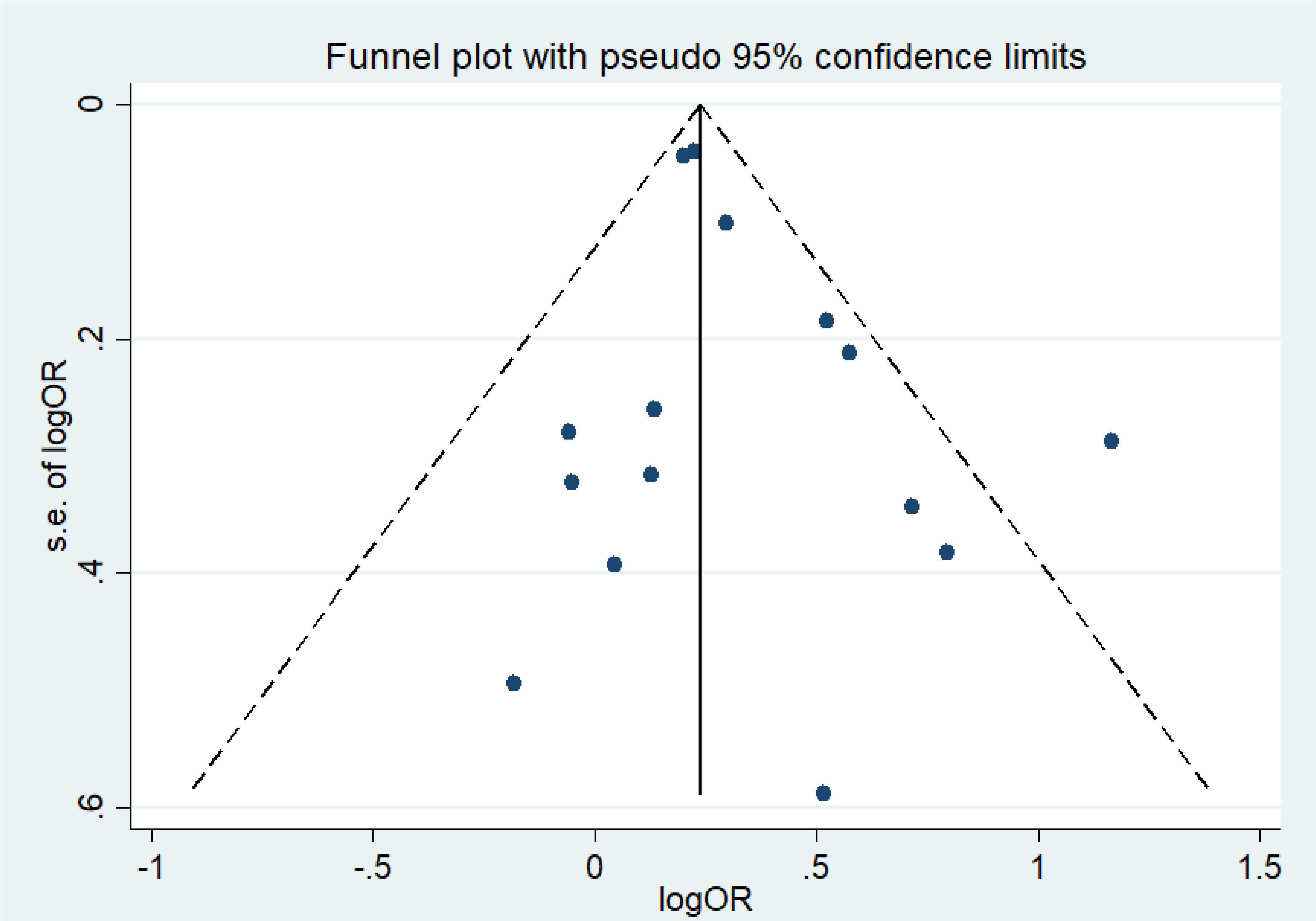

eFigure 3b Funnel plot for female gender

© 2020 Zhou AY et al. *JAMA Network Open.*

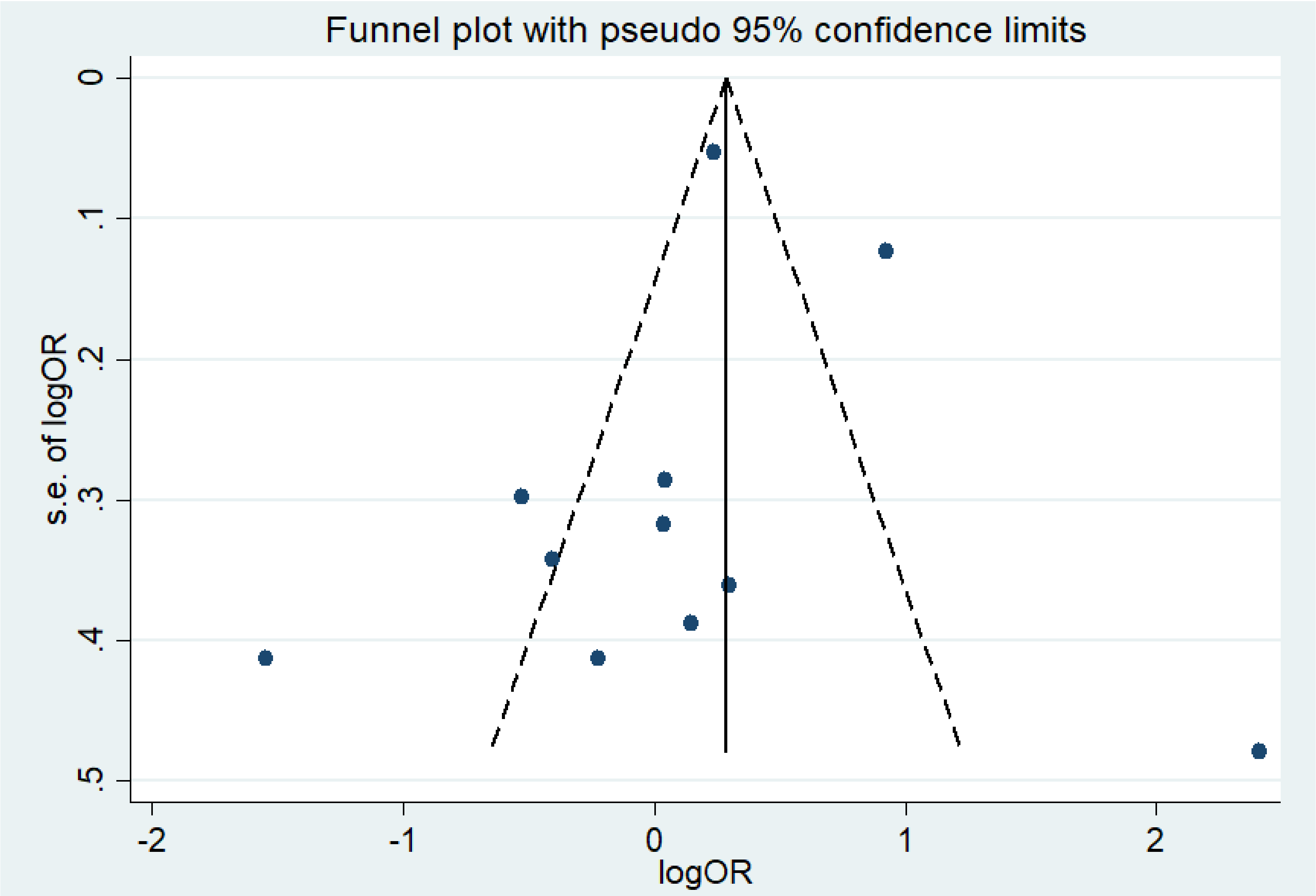

eFigure 3c Funnel plot for more junior grade

© 2020 Zhou AY et al. *JAMA Network Open.*

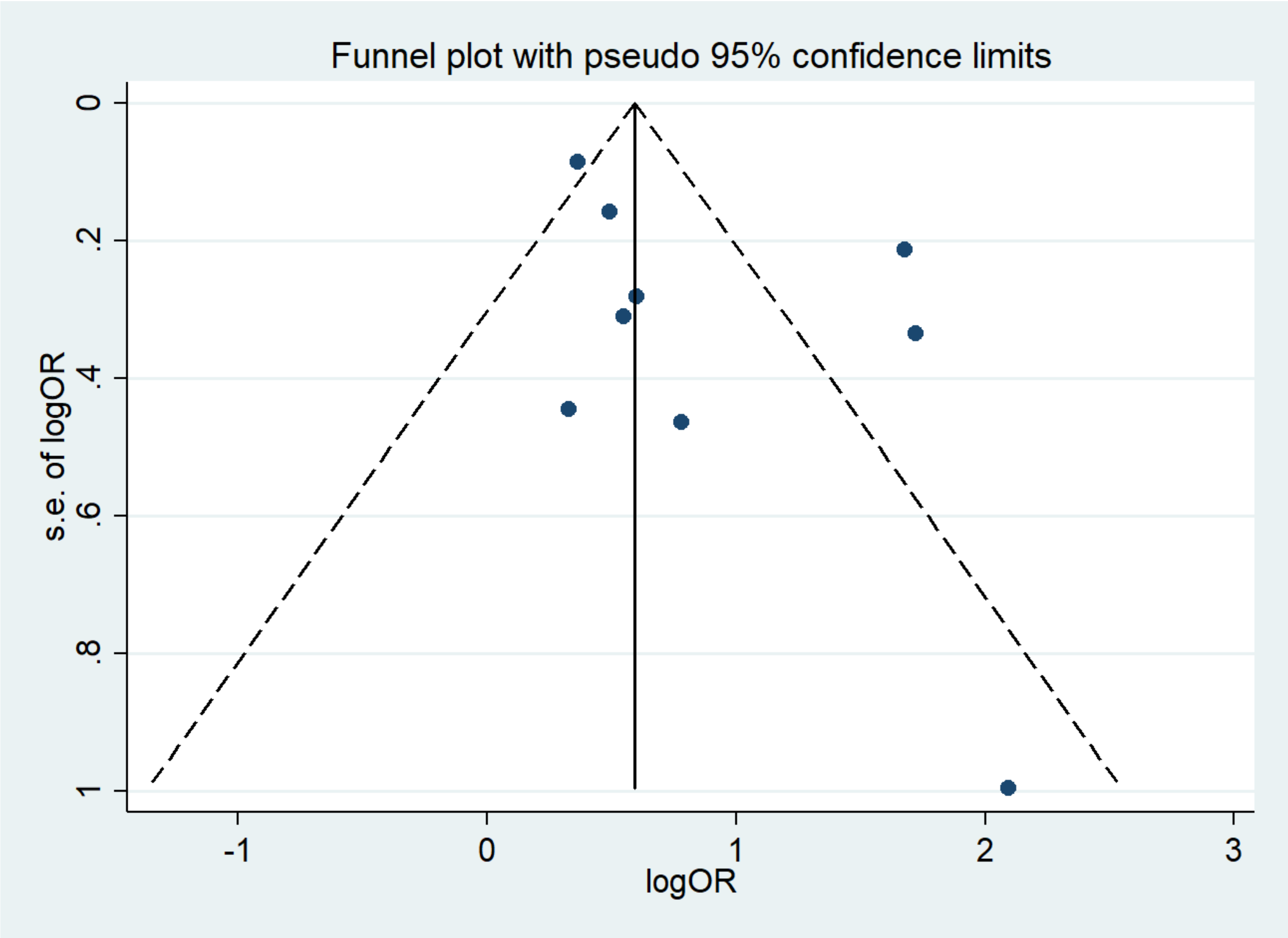

eFigure 3d Funnel plot for concerns about patient care

© 2020 Zhou AY et al. *JAMA Network Open.*

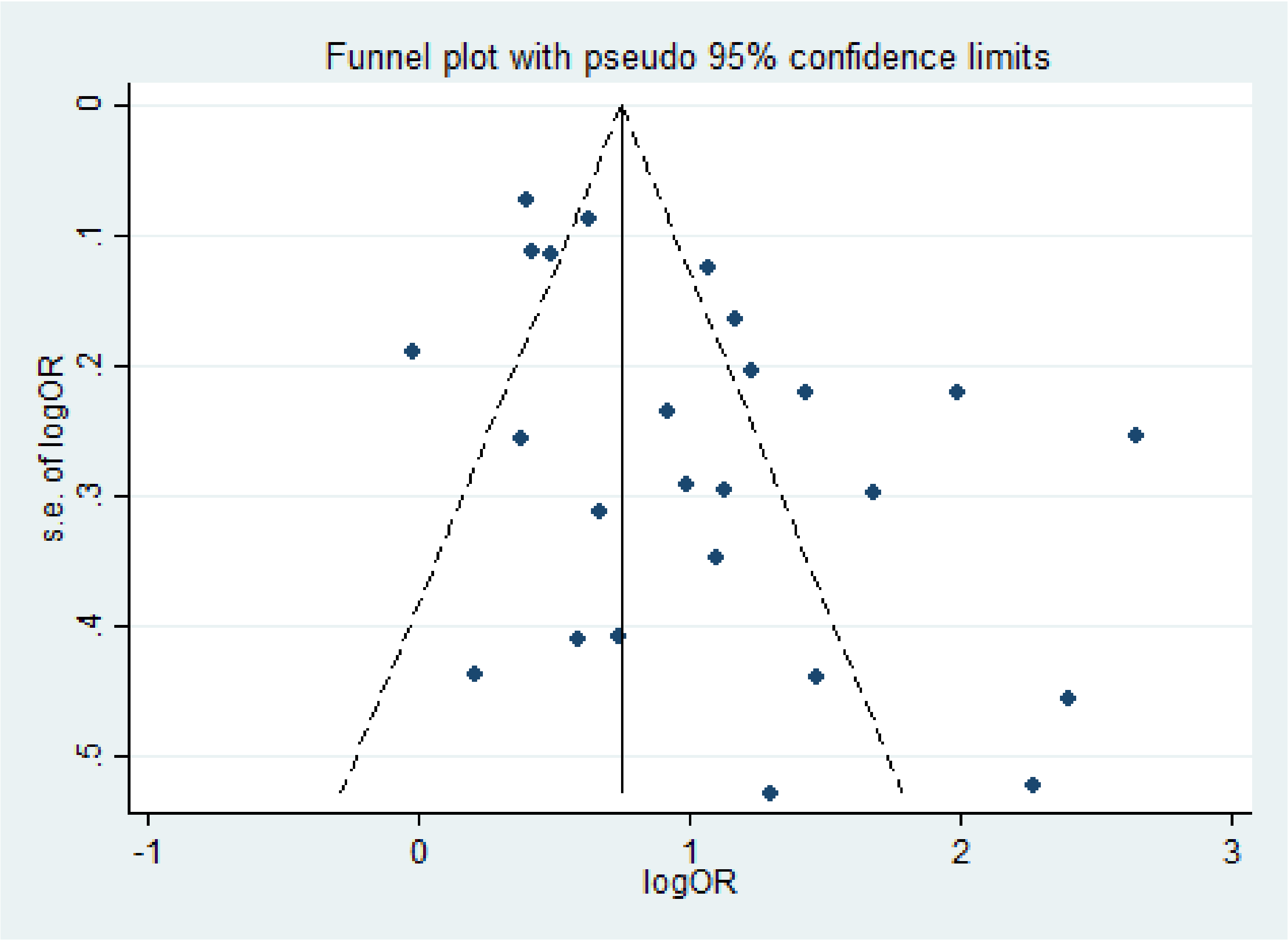

eFigure 3e Funnel plot for work demands

© 2020 Zhou AY et al. *JAMA Network Open.*

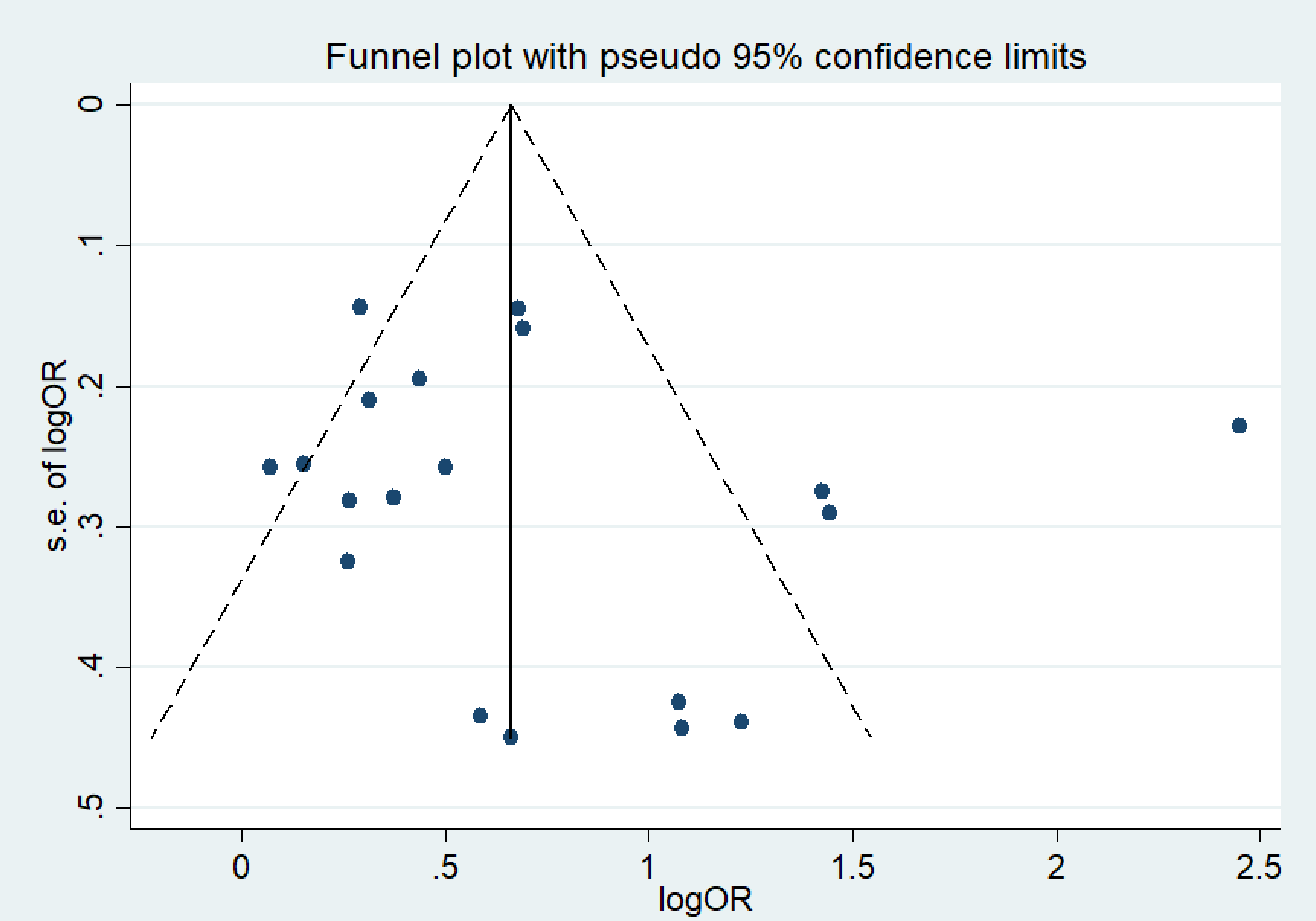

eFigure 3f Funnel plot for work environment

© 2020 Zhou AY et al. *JAMA Network Open.*

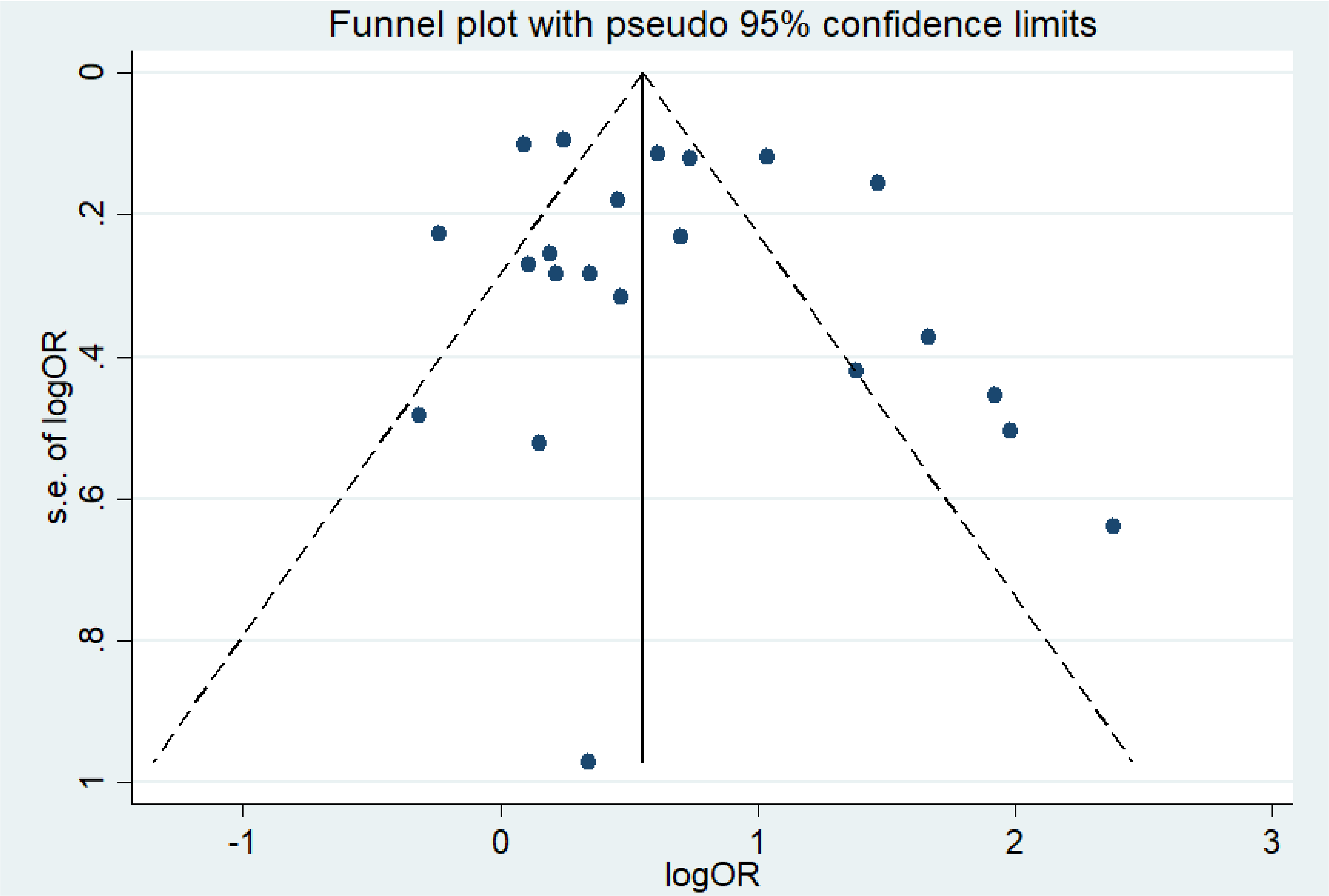

eFigure 3g Funnel plot for work-life balance

© 2020 Zhou AY et al. *JAMA Network Open.*

eMethods systematic review protocol

Stressors in Trainee Doctors and its relation to burnout and distress: A Systematic Review and meta-analysis

Protocol

Background

Trainee doctors are fully qualified doctors engaged in postgraduate training(1). There is solid evidence that doctors experience excessive levels of stress (distress) and burnout, and that trainee doctors are a particularly high-risk group. (2-5). Burnout consists of three components: emotional exhaustion, reduced sense of personal achievement as well as depersonalisation (6), and one of its main contributors is prolonged occupational stress (7). High levels of burnout and stress have been found in trainee doctors working in the US and other countries such as Australia and Canada (8-12). This was also mirrored in a recent national survey of 51956 trainee doctors working in the United Kingdom (UK) has found that nearly a quarter of trainee doctors were experiencing burnout to a concerning degree. Another survey conducted by Health Education England found that 50% of trainees were experiencing symptoms suggestive of burnout and 80% of trainees felt their job caused excessive stress (13). Burnout has negative impact on the personal wellbeing, the family and professional relationships and the career prospects of trainee doctors and may jeopardise patient care. The wellbeing of trainee doctors is a key benchmark for the efficiency and sustainability of healthcare systems in this and the following decades (14, 15).

Several studies have identified a wide range of contributors to stress (stressors) which have been associated with distress and burnout in trainee doctors. Examples of stressors include personal characteristics such as female gender as well as occupational stressors such as workload, financial worries and work-life conflict (11,40,47,53,59). However, variations in methodology and presentation of results across studies make it difficult to compare the results between the studies and to explore the consistencies and inconsistencies of the effects. Thus, a major limitation in the current evidence base of stressors contributing to burnout in trainee doctors is the absence of an evidence synthesis to present the results using common metrics and draw rigorous conclusions and research and clinical directions.

© 2020 Zhou AY et al. *JAMA Network Open.*

It is to our knowledge that no previous systematic reviews have been identified in the scientific literature and we did not identify any similar systematic reviews on the PROSPERO database. Hence, we propose to conduct a systematic review and meta-analysis that aims to identify occupational and non-occupational stressors that are associated with burnout/distress in trainee doctors.

We will use methods reported in line with the Preferred Reporting Items for Systematic Reviews and Meta-Analyses (PRISMA) guidelines (16) as well as Meta-analysis Of Observational Studies in Epidemiology (MOOSE) (17). We will also present the results for this review in line with the PRISMA and MOOSE guidelines.

Search Strategy

We plan to search the following databases:

- Medline
- Embase
- Psych INFO
- Cochrane Database of Systematic reviews

We will search for eligible papers from inception until July 2018. The search strategy will include the following combinations of three key blocks of terms:

- Stress/ burnout
- trainee doctors
- determinants of stress/stressors

We will use a combination of Medical Subject Headings and text-words and our search strategy can be found in figure 1. In order to try and capture all trainee doctors as a group, used a wide range of nomenclature for trainee doctors in our search and this included terms such as trainee, foundation year, registrar, resident, intern and specialty trainee (eTable 3).

© 2020 Zhou AY et al. *JAMA Network Open.*

1	distress*
2	nervous*
3	Stress, Psychological/ or stress*
4	Anxiety/ or anxiety
5	apprehension
6	worry*
7	Burnout, Professional/ or burnout*
8	or/1-7
9	((trainee or trainee or foundation year or registrar or resident) adj2 (physician* or doctor*))
10	(speciality trainee* or intern)
11	Physicians/
12	(trainee or trainee or foundation year or registrar)
13	11 and 12
14	9 or 10 or 13
15	(determinant* or factor* or driver* or caus* or contributor* or stressor* or predictor* or predispos* or correlat* or associat* or risk*)
16	8 and 14 and 15

eTable 3. Search strategy

Database searches will be supplemented by hand searches of reference lists of included papers.

© 2020 Zhou AY et al. *JAMA Network Open.*

Eligibility Criteria

Studies were eligible for inclusion if they met the following criteria:

- Population: Qualified doctors who are engaged in postgraduate training (i.e. trainee doctors). Studies that are based on a mix of trainee doctors and other doctors or health professionals were included if trainee doctors comprised of at least 70% of the sample.
- Stressors: Stressors can lead to stress which in turn can lead to distress and burnout. Stressors will include occupational and non-occupational contributors of stress such as work demands, specialty, work environment and demographics.
- Outcome: The main outcomes will be associations between stressors (occupational and non-occupational contributors of stress) and negative outcomes of stress such as burnout/distress. We will include both burnout measured with validated measures such as the Maslach Burnout Inventory as well as measures of distress because both are known negative outcomes of stress (15). A pooled analysis of burnout and distress will be undertaken. A separate analysis will be undertaken on burnout to examine burnout as an outcome of prolonged stress.
- Design: Quantitative research design such as observational studies including retrospective or prospective cohort and cross-sectional as well as case control studies.
- Context: Any healthcare setting including primary and secondary care.

Exclusion Criteria

- Studies not explicitly focusing on stress such as studies that explore the determinants of psychiatric conditions with specific diagnostic criterion e.g. depression and generalised anxiety disorder.
- Grey literature, conference abstracts and letters to the editor and studies not published in a peer-reviewed journal were excluded.
- Studies not written in English

© 2020 Zhou AY et al. *JAMA Network Open.*

Study Selection

Searches will be exported onto Endnote and duplicate will be removed. Study selection will be completed in two stages. Firstly, the titles and abstracts of the identified studies will be screened and subsequently the full-texts of relevant studies were accessed and further screened against the eligibility criteria. Both MP and AZ will be involved in the screening. Disagreements will be resolved through discussions.

Data extraction

Data extraction will be done in an excel form and this will be initially piloted on 5 randomly selected studies. The following descriptive data will be extracted:

- Study: country, method of recruitment, healthcare setting, clinical code (primary care, hospitals or mixed), research design, control, location
- Population: sample size, age, gender, specialty, grade of trainee doctor
- Outcomes: Outcome of stressors (burnout, emotional distress, other), types of analysis used, type of stressors identified.

Methodological quality of the studies

We will use the Newcastle Ottawa scale to critically appraise the quality of the included studies (18) and will use the adapted version to undertake critical appraisal assessments of cross-sectional studies (19). This modified Newcastle Ottawa instrument shown below (Fig. 1) provides scores from 0 to 10 with studies scoring ≥6 classified as high quality.

© 2020 Zhou AY et al. *JAMA Network Open.*

Newcastle-Ottawa Scale adapted for cross-sectional studies

Selection: (Maximum 5 stars)

1) Representativeness of the sample:

a) Truly representative of the average in the target population. * (all subjects or random sampling)

b) Somewhat representative of the average in the target population. * (non-random sampling)

c) No description of the sampling strategy.

2) Selected group of users

a) Due to relevant selection of individuals to exclude factors that will bias results (exclusion criteria? account for non-respondents? Did the authors justify selection?) *

b) No relevant/systematic selection

3) Sample size:

a) Justified and satisfactory (power calculation included). *

b) Not justified.

4) Ascertainment of exposure i.e. stress/burnout

a) Secure records (e.g. medical records) *

b) Structured interview *

c) written self-report

d) no description

Comparability: (Maximum 2 stars)

1) The subjects in different outcome groups are comparable, based on the study design or analysis. Confounding factors are controlled.

a) The study controls for different independent factors**

b) The study controls for only 1 factor. *

c) no confounding factors are controlled

Outcome: (Maximum 3 stars)

1) Ascertainment of the method (e.g. to measure stress/burnout):

a) Validated measurement method **

b) Non-validated measurement method, but the method is available or described.*

c) No description of the measurement tool.

2) Statistical test:

a) The statistical test used to analyze the data is clearly described and appropriate, and the measurement of the association is presented (including SD/SE, probability level (p value)). *

b) The statistical test is not appropriate, not described or incomplete.

OUT OF 10:

Fig. 1 Adapted Newcastle Ottawa Scale proforma for cross sectional studies

© 2020 Zhou AY et al. *JAMA Network Open.*

Data Analysis

The primary outcome of this review will be the association of stressors with burnout/distress in trainee doctors. We will calculate odds ratios (ORs) together with the 95% confidence intervals from each study using Comprehensive Meta-analysis (CMA) software (20). The pooled ORs and the forest plots will be computed using the metaan command in STATA (21). We will use ORs to pool the results because this was the most commonly reported estimated for effect in individual studies and because ORs are considered more appropriate for cross sectional studies compared with other estimates such as relative risk (22). In this study, OR>1 indicates that the stressor is associated with an increased risk of burnout or distress, whereas OR<1 indicates that the stressor is associated with a decrease risk. In accordance to recommendations (20), across studies reporting multiple measures of the same stressor category (e.g. different measures of job demands such as on call or long working hours), the median ORs will ve computed to ensure that each study contributed only one effect measure to each meta-analysis. The I^2 statistic will be used to assess heterogeneity among studies. Conventionally, I^2 values of 25%, 50% and 75% indicate low, moderate and high heterogeneity respectively (23).

Two sensitivity analyses will be performed to examine whether the results are stable when only i) highly rated methodologically studies were retained in the analyses (a score of ≥6) and ii) studies using measures of burnout only. Including measures of only burnout would enable us to assess whether the results are stable when only prolonged stress (burnout) outcomes are included.

Potential for publication bias will be assessed on all pooled outcomes which included ≥9 studies (24). The possibility of publication bias will be examined by inspecting the symmetry of funnel plots and using Egger's test (25). Funnel plots were constructed using the metafunnel command and the Egger test was computed using the metabias command (26, 27). All analyses will be performed in Stata version 14.

© 2020 Zhou AY et al. *JAMA Network Open.*

eReferences

1. Association. BM. Doctors' titles: explained. 2017.
2. Shanafelt TD, Boone S, Tan L, Dyrbye LN, Sotile W, Satele D, et al. Burnout and satisfaction with work-life balance among US physicians relative to the general US population. Arch Intern Med. 2012;172(18):1377-85.
3. Peisah C, Latif E, Wilhelm K, Williams B. Secrets to psychological success: why older doctors might have lower psychological distress and burnout than younger doctors. Aging Ment Health. 2009;13(2):300-7.
4. Stucky ER, Dresselhaus TR, Dollarhide A, Shively M, Maynard G, Jain S, et al. Intern to attending: assessing stress among physicians. Acad Med. 2009;84(2):251-7.
5. Dyrbye LN, West CP, Satele D, Boone S, Tan L, Sloan J, et al. Burnout among U.S. medical students, residents, and early career physicians relative to the general U.S. population. Acad Med. 2014;89(3):443-51.
6. Maslach C, Jackson SE, Leiter MP, Schaufeli WB, Schwab RL. Maslach burnout inventory: Consulting Psychologists Press Palo Alto, CA; 1986.
7. Maslach C, Schaufeli WB, Leiter MP. Job burnout. Annual review of psychology. 2001;52(1):397-422.
8. Markwell AL, Wainer Z. The health and wellbeing of junior doctors: insights from a national survey. The Medical journal of Australia. 2009;191(8):441-4.
9. Cohen JS, Patten S. Well-being in residency training: a survey examining resident physician satisfaction both within and outside of residency training and mental health in Alberta. BMC medical education. 2005;5:21.
10. Abdulghani HM, Al-Harbi MM, Irshad M. Stress and its association with working efficiency of junior doctors during three postgraduate residency training programs. Neuropsychiatric disease and treatment. 2015;11:3023-9.
11. Haoka T, Sasahara S, Tomotsune Y, Yoshino S, Maeno T, Matsuzaki I. The effect of stress-related factors on mental health status among resident doctors in Japan. Med Educ. 2010;44(8):826-34.
12. Ogunsemi OO, Alebiosu OC, Shorunmu OT. A survey of perceived stress, intimidation, harassment and well-being of resident doctors in a Nigerian Teaching Hospital. Nigerian journal of clinical practice. 2010;13(2):183-6.
13. England HE. Junior doctor morale: Understanding best practice working environments. 2017.
14. Prins JT, Gazendam-Donofrio SM, Tubben BJ, van der Heijden FM, van de Wiel HB, Hoekstra-Weebers JE. Burnout in medical residents: a review. Med Educ. 2007;41(8):788-800.
15. West CP, Dyrbye LN, Shanafelt TD. Physician burnout: contributors, consequences and solutions. J Intern Med. 2018;283(6):516-29.
16. Liberati A, Altman DG, Tetzlaff J, Mulrow C, Gotzsche PC, Ioannidis JP, et al. The PRISMA statement for reporting systematic reviews and meta-analyses of studies that evaluate healthcare interventions: explanation and elaboration. BMJ. 2009;339:b2700.
17. Stroup DF, Berlin JA, Morton SC, Olkin I, Williamson GD, Rennie D, et al. Meta-analysis of observational studies in epidemiology: a proposal for reporting. Meta-analysis Of Observational Studies in Epidemiology (MOOSE) group. JAMA. 2000;283(15):2008-12.
18. Wells G, Shea B, O'connell D, Peterson J, Welch V, Losos M, et al. The Newcastle-Ottawa Scale (NOS) for assessing the quality of nonrandomised studies in meta-analyses. Ottawa Hospital Research Institute, 2014. oxford. ASp; 2015.
19. Hygum K, Starup-Linde J, Harsløf T, Vestergaard P, Langdahl BL. Mechanisms in endocrinology: Diabetes mellitus, a state of low bone turnover–a systematic review and meta-analysis. European journal of endocrinology. 2017;176(3):R137-R57.
20. Borenstein M, Hedges LV, Higgins JP, Rothstein HR. Introduction to meta-analysis: John Wiley & Sons; 2011.
21. Kontopantelis E, Reeves D. metaan: Random-effects meta-analysis. Stata Journal. 2010;10(3):395.
22. Deeks JJ HJ, Altman DG. 9.2 Types of data and effect measures. 2011. In: Cochrane Handbook for Systematic Reviews of Interventions Version 510. The Cochrane Collaboration.
23. Higgins JP, Thompson SG, Deeks JJ, Altman DG. Measuring inconsistency in meta-analyses. BMJ: British Medical Journal. 2003;327(7414):557.
24. Sterne JA, Gavaghan D, Egger M. Publication and related bias in meta-analysis: power of statistical tests and prevalence in the literature. Journal of clinical epidemiology. 2000;53(11):1119-29.
25. Egger M, Smith GD, Schneider M, Minder C. Bias in meta-analysis detected by a simple, graphical test. Bmj. 1997;315(7109):629-34.
26. Sterne JA, Harbord RM. Funnel plots in meta-analysis. Stata Journal. 2004;4:127-41.
27. Harbord RM, Harris RJ, Sterne JA. Updated tests for small-study effects in meta-analyses. Stata Journal. 2009;9(2):197.

© 2020 Zhou AY et al. *JAMA Network Open.*
